# Synchronous Telemedicine Versus In‐Person Care in Hepatitis C Treatment: A Systematic Review and Meta‐Analysis

**DOI:** 10.1111/jvh.70144

**Published:** 2026-01-28

**Authors:** Igor Boechat Silveira, Gustavo Procópio Silva, Arthur Victor de Holanda Sampaio, Milena Ramos Tomé, Jingying Elena Chen, Alana Vitória Santos de Jesus, Guilherme Grossi Lopes Cançado

**Affiliations:** ^1^ Universidade Federal de Juiz de Fora Juiz de Fora Brazil; ^2^ Universidade Federal de Roraima Roraima Brazil; ^3^ Universidade Federal de Alagoas Maceió Brazil; ^4^ Universidade Federal de Campina Grande Cajazeiras Brazil; ^5^ Universidade Idomed Vista Carioca Rio de Janeiro Brazil; ^6^ Universidade Federal da Bahia Salvador Brazil; ^7^ Hospital das Clínicas da Universidade Federal de Minas Gerais Belo Horizonte Brazil; ^8^ Hospital da Polícia Militar de Minas Gerais Belo Horizonte Brazil

**Keywords:** hepatitis C, meta‐analysis, systematic review, telemedicine

## Abstract

Inequitable access to HCV treatment persists, particularly for rural and marginalised populations. Synchronous telemedicine (TM) could mitigate access barriers, but its comparative effectiveness versus in‐person care is uncertain. We performed a systematic review and meta‐analysis comparing synchronous TM with in‐person care for HCV. The primary outcome was sustained virologic response (SVR); secondary outcomes were treatment initiation and completion. Subgroup analyses examined study design, therapy era (interferon vs. direct‐acting antivirals [DAAs]), and setting (rural vs. non‐rural). Narrative synthesis addressed people who use drugs (PWUD), incarcerated populations, pandemic‐era cohorts, and economic evaluations. Fifteen studies involving 7.459 patients (2 RCTs, 13 observational) were included (13 meta‐analysed). For SVR, the pooled effect showed no significant difference between interventions (odds ratio [OR] 1.60, 95% CI 0.69–3.68). Treatment initiation and completion were also not significantly different overall (initiation OR 7.59, 95% CI 0.79–72.81; completion OR 2.50, 95% CI 0.76–8.25), although exclusion of single influential studies yielded significant benefits for TM in sensitivity analyses. Subgroups suggested context‐specific advantages: TM favoured SVR in rural settings (OR = 4.19, 95% CI 1.28–13.73) and in RCTs (OR = 10.42, 95% CI 7.41–14.67). Narrative evidence indicated that TM improved linkage and cure among PWUD and incarcerated individuals, preserved efficacy during COVID‐19, and reduced costs. Overall, synchronous TM seems comparable to in‐person care overall and may be superior in rural and marginalised populations.

**Abbreviations**
AASLD: American Association for the Study of Liver Diseases
CENTRAL: Cochrane Central Register of Controlled Trials
CI: Confidence Interval
DAAs: direct‐acting antivirals
DBS: Dried Blood Spot
DL: DerSimonian‐Laird
EUC: enhanced usual care
HCC: hepatocellular carcinoma
HCV: hepatitis C virus
HMP: Her Majesty's Prison
ID: Infectious Disease
IDSA: Infectious Diseases Society of America
IFN: interferon
ITT: intention‐to‐treat
MD: Doctor of Medicine
MH: Mantel–Haenszel
NHS: National Health Service
OR: odds ratio
PhD: Doctor of Philosophy
PRISMA: Preferred Reporting Items for Systematic Reviews and Meta‐Analyses
PROSPERO: International Prospective Register of Systematic Reviews
PWUD: people who use drugs
RCTs: randomised controlled trials
RNA: Ribonucleic Acid
RoB 2: Cochrane Risk of Bias 2 (tool)
ROBINS‐I: Risk of Bias in Non‐Randomised Studies of Interventions (tool)
RR: relative risk
SVR: sustained virologic response
TM: telemedicine
UK: United Kingdom
USA: United States of America
WHO: World Health Organisation

## 1 Introduction

Hepatitis C virus (HCV) infection is a major cause of chronic liver disease worldwide. While often asymptomatic in its early stages, persistent infection leads to progressive fibrosis, cirrhosis, and hepatocellular carcinoma (HCC). The resulting morbidity and mortality remain substantial, particularly as the burden of HCV‐related cirrhosis and HCC continues to rise [1, 2]. Globally, approximately 1 million new cases of chronic HCV infection are diagnosed each year [3, 4]. In 2022, the World Health Organisation (WHO) reported that hepatitis C was responsible for 242,000 deaths worldwide, with nearly 5 million people affected in the Americas alone. To address this burden, the WHO has set ambitious elimination targets: reducing new hepatitis infections to 520,000 annually and decreasing hepatitis‐related deaths by 65% by 2030 [3, 5, 6].

Treatment of HCV has been revolutionised by the advent of direct‐acting antivirals (DAAs), which achieve sustained virologic response (SVR) rates exceeding 95% with a favourable safety profile, surpassing those of interferon (IFN)‐based regimens. Despite these advances, the major barrier to elimination remains inequitable access to diagnosis and treatment, which limits the full population‐level impact of DAAs [2, 3, 5]. This challenge disproportionately affects marginalised populations, including people experiencing homelessness, incarcerated individuals, rural communities, and people who use drugs (PWUD), who face multiple barriers across the HCV care cascade—screening, diagnosis, linkage to care, and treatment [5, 7, 8].

Telemedicine (TM) has emerged as a promising strategy to expand access to HCV care, particularly in rural, underserved, and correctional settings. Teleconsultation programs have demonstrated SVR rates equivalent to those achieved through in‐person care, without increasing treatment failure and often reporting higher levels of patient satisfaction [7, 9]. By enabling primary care providers to initiate treatment under specialist guidance, TM decentralises HCV management, increases system capacity, and reduces geographic, logistical, and structural disparities in access to care [7].

This systematic review and meta‐analysis aims to synthesise the available evidence on the role of synchronous TM models (video/phone visits) rather than asynchronous or purely diagnostic uses in the management of chronic HCV infection, with the primary objective of comparing synchronous teleconsultation with face‐to‐face care in terms of SVR.

## 2 Materials and Methods

### 2.1 Protocol Registration

This systematic review and meta‐analysis was conducted in accordance with the *Preferred Reporting Items for Systematic Reviews and Meta‐Analyses* (PRISMA) guidelines [10, 11] and the *Cochrane Handbook for Systematic Reviews of Interventions* [12]. The study protocol was registered in PROSPERO (CRD420251133988). The PRISMA Guidelines Checklist is provided in Table S1.

### 2.2 Eligibility Criteria

We included studies that met the following criteria: (1) adult patients (≥ 18 years) with chronic or active hepatitis C virus (HCV) infection; (2) use of a synchronous TM intervention for care delivery, defined as real‐time, two‐way communication between a patient and a healthcare provider—primarily through video conferencing or live phone calls—covering remote consultations, prescription management, and HCV treatment follow‐up; (3) reporting of at least one outcome of interest; (4) inclusion of a comparison group receiving in‐person standard care with the same prescribed therapy as the intervention group; (5) randomised controlled trials or observational cohort studies published in English in peer‐reviewed journals.

Exclusion criteria were: (1) interventions limited to asynchronous telehealth methods (e.g., email, text messaging) without a synchronous component; (2) TM used solely for diagnostic or screening purposes rather than treatment delivery; (3) overlapping study populations; or (4) case reports, case series, systematic reviews, meta‐analyses, conference abstracts, or studies not reporting outcomes of interest.

### 2.3 Search Strategy and Data Extraction

We systematically searched PubMed (MEDLINE), Embase, and the Cochrane Central Register of Controlled Trials (CENTRAL) from inception to June 25, 2025. Reference lists of eligible articles and relevant systematic reviews or meta‐analyses were also screened to identify additional studies. Our search used terms presented in a previously validated strategy [13], detailed in Table S2. Following deduplication (I.S.), two reviewers (I.S. and A.S.) independently screened titles and abstracts, with potentially relevant studies undergoing full‐text review. Bibliographic management was performed using Rayyan [14]. Data extraction was conducted by one reviewer (I.S.) using a structured spreadsheet that included the following domains: study title, journal, year, first author, country, TM definition, inclusion criteria, intervention description, comparator characteristics, treatment type (interferon‐based vs. DAA‐based), follow‐up duration, geographical and social context, sample size, age, sex, liver fibrosis assessment, HCV genotype, and reported outcomes. A second reviewer (G.S.) verified all extracted data.

### 2.4 Endpoints

The primary outcome was sustained virologic response (SVR). Secondary outcomes included: (1) treatment completion; (2) treatment initiation; and (3) comparison of baseline characteristics between synchronous TM and in‐person care groups. Subgroup analyses were performed according to (1) therapy type (interferon‐based vs. direct‐acting antivirals); (2) geographical setting (rural vs. non‐rural); and (3) study design (RCTs vs. observational studies).

In addition, we evaluated a set of exploratory endpoints aimed at capturing broader dimensions of HCV care that may affect the effectiveness and scalability of TM. These included (1) feasibility, adherence, and treatment outcomes among PWUD; (2) the effectiveness of TM in correctional settings for incarcerated people; (3) the impact of COVID‐19 on access to care, treatment delivery, and SVR ascertainment during the pandemic‐driven shift to TM; and (4) the economic aspects of TM, including cost‐effectiveness, cost‐savings, and resource utilisation.

### 2.5 Quality Assessment

Two reviewers (I.S. and R.R.) independently assessed study quality. Risk of bias for randomised studies was evaluated using the Cochrane *Risk of Bias 2* (RoB 2) tool [15], while non‐randomised studies were assessed using version 2 of the Risk of Bias in Non‐Randomised Studies of Interventions (ROBINS‐I) tool [16]. Disagreements were resolved by consensus with a third author (G.S.). Traffic‐light plots were generated for both tools. Publication bias was assessed only for the primary outcome, as a minimum of 10 studies is required for this analysis. We used a two‐part approach: a qualitative inspection of the funnel plots and a quantitative analysis using the Begg–Mazumdar rank continuity correlation and Egger's regression tests [17, 18].

### 2.6 Statistical Analysis

All analyses were conducted in R (version 4.4.3) using the meta and metafor packages. Treatment effects for binary outcomes were expressed as odds ratios (ORs) and calculated using the Mantel–Haenszel (MH) method. Between‐study variance was estimated with the DerSimonian‐Laird (DL) method for confidence intervals. Statistical heterogeneity was assessed with the *I* ^2^ statistic and classified as minimal (0%–40%), moderate (30%–60%), substantial (50%–90%), or considerable (75%–100%) [19]. A random‐effects model was applied in all analyses. For any given outcome, a minimum of two studies was required to generate a forest plot. Results are presented graphically as forest plots.

Sensitivity analysis for meta‐analysis comprising at least 5 trials was performed with the leave‐one‐out method to assess the robustness of the summary estimates. This threshold was chosen because such analyses become statistically unstable and less informative when applied to a smaller number of trials, considering the removal of any one study would disproportionately affect the pooled estimate.

## 3 Results

Our search identified 1391 articles. After removing duplicates (*n* = 397) and excluding studies based on title and abstract screening (*n* = 919), 75 full‐text articles were assessed for eligibility. Of these, 15 studies met the inclusion criteria, and 13 provided sufficient data for meta‐analysis. The PRISMA flowchart is presented in Figure S1.

### 3.1 Study Characteristics

A total of 7459 patients were included across the 15 studies, with 1705 receiving TM care and 5754 receiving standard in‐person care. Ten studies were conducted in North America (eight in the United States, two in Canada), three in Europe (two in Spain, one in England), one in Taiwan, and one in Australia. Two were randomised controlled trials (RCTs), while 13 were observational cohort studies. The studies by Cooper et al. [20] and Lepage et al. [21] involved overlapping populations; therefore, our analysis only included the study that reported the specific outcome of interest.

The included study populations exhibited significant heterogeneity regarding clinical status, social determinants of health, and geographical setting. General clinic‐based populations were evaluated in five studies [20, 21, 22, 23, 24], which broadly included adults aged 18 or older referred for HCV therapy or engaged with hospital‐based viral hepatitis programs. Studies conducted during the interferon era [25, 26] applied stricter clinical inclusion criteria, requiring participants to be treatment‐naïve, have elevated alanine aminotransferase (ALT) levels, or biopsy‐proven chronic infection.

A substantial proportion of studies targeted specific vulnerable or hard‐to‐reach populations. Rural cohorts were explicitly examined by Case et al. [27] and Nazareth et al. [28], who included rural patients with compensated hepatitis C (non‐cirrhotic and cirrhotic). PWUD were the primary focus for Talal et al. [29] (patients enrolled in opioid treatment programs), Morales‐Arraez et al. [30] (attendees of drug treatment centers), and Seaman et al. [31] (rural persons who inject drugs). Additionally, Trammel et al. [32] evaluated a unique cohort of pregnant PWUD patients. Finally, incarcerated populations were assessed by Morey et al. [33] and Cuadrado et al. [34], whose inclusion criteria were limited to inmates with confirmed viremia in specific prison systems. Key study characteristics are summarised in Table 1, while Table S3 provides detailed information on each study's definitions for telemedicine, SVR, and inclusion criteria.

**TABLE 1:** Characteristics of the studies included in the systematic review and meta‐analysis.

Study (year)	Design	Country	Type of therapy	Total of patients: TM/UC	Male, *n* (%): TM/UC	Mean/median age (years): TM/UC	White, *n* (%) TM/UC	Follow‐up, months
Case et al. (2019)	non‐RCT	USA	DAA and IFN	135/629	131 (95.0)/636 (97.0)	62/62	117 (85.0)/428 (65.0)	6
Chen et al. (2014)	non‐RCT	Taiwan	IFN	148/150	NA/NA	51.5/46.5	NA/NA	18
Cooper et al. (2017)	non‐RCT	USA	DAA	274/1230	155 (56.5)/695 (56.5)	38/41	244 (89.1)/1114 (90.6)	4
Cooper et al. (2022)	non‐RCT	Canada	DAA	157/1230	104 (66.2)/716 (63.4)	48.1/49.0	118 (75.2)/770 (68.1)	4
Cuadrado et al. (2021)	non‐RCT	Spain	DAA	75/0^a^	NA/NA	NA/NA	NA/NA	13
Frye et al. (2023)	non‐RCT	USA	DAA	99/388	177 (70.2)/258 (66.5)	58.9/62.1	46 (18.3)/65 (16.8)	3
Lepage et al. (2020)	non‐RCT	Canada	DAA and IFN	106/1267	72 (67.9)/804 (63.8)	48.2/49.3	74 (69.8)/877 (69.2)	4
Morales‐Arraez et al. (2021)	non‐RCT	Spain	DAA	32/37	30 (93.8)/29 (78.4)	47.1/48.9	NA/NA	24
Morey et al. (2019)	non‐RCT	England	DAA	80/4	80 (100.0)/4 (100.0)	NA/NA	NA/NA	27
Nazareth et al. (2013)	non‐RCT	Australia	IFN	50/279	25 (50.0)/25 (50.0)	46/NA	NA/NA	24–48
Rossaro et al. (2013)	non‐RCT	USA	IFN	40/40	19 (47.5)/22 (55.0)	51/53	29 (72.5)/24 (60.0)	6
Seaman et al. (2025)	RCT	USA	DAA	100/103	58 (58.0)/68 (66.0)	41.0/42.2	89 (89.0)/90 (87.0)	12
Talal et al. (2024)	RCT	USA	DAA	290/312	175 (60.3)/194 (62.2)	47.1/48.9	155 (53.4)/151 (48.4)	24
Trammel et al. (2024)	non‐RCT	USA	DAA	42/29	NA/NA	30/29	33 (79.0)/24 (83.0)	1.5–3
Yang et al. (2025)	non‐RCT	USA	DAA	77/56	41 (53.2)/45 (80.4)	47.1/48.9	53 (68.8)/29 (51.8)	12

Abbreviations: DAA, Direct‐Acting Antivirals; NA, Not Available; RCT, Randomised Controlled Trial; TM, Telemedicine; UC, Usual Care; USA, United States of America.

^a^ The usual care group was not a separate, concurrent cohort of patients. It was a retrospectively modelled simulation based on the same 75 inmates who received telemedicine. The model calculated the hypothetical costs and resources these patients would have incurred if their consultations had been traditional face‐to‐face visits.

### 3.2 Risk of Bias

Risk of bias was assessed separately for randomised and non‐randomised studies. The two randomised controlled trials [29, 31], evaluated with the RoB 2 tool, were both judged to have an overall low risk of bias across all domains. In contrast, the thirteen non‐randomised studies [20, 21, 22, 23, 24, 26, 27, 28, 30, 32, 33, 34, 35], assessed with the ROBINS‐I tool, demonstrated considerable methodological concerns. Six of these studies were rated at a serious overall risk of bias and two at a moderate risk, with no study achieving a low‐risk rating. The primary sources of bias in this group were consistently related to confounding and participant selection, although the risk was generally low for domains concerning intervention classification, outcome measurement, and reporting. Overall judgements are presented in Figures S2 and S3.

### 3.3 Meta‐Analysis

#### 3.3.1 Primary Outcome: Sustained Virologic Response (SVR)

**FIGURE 1:**
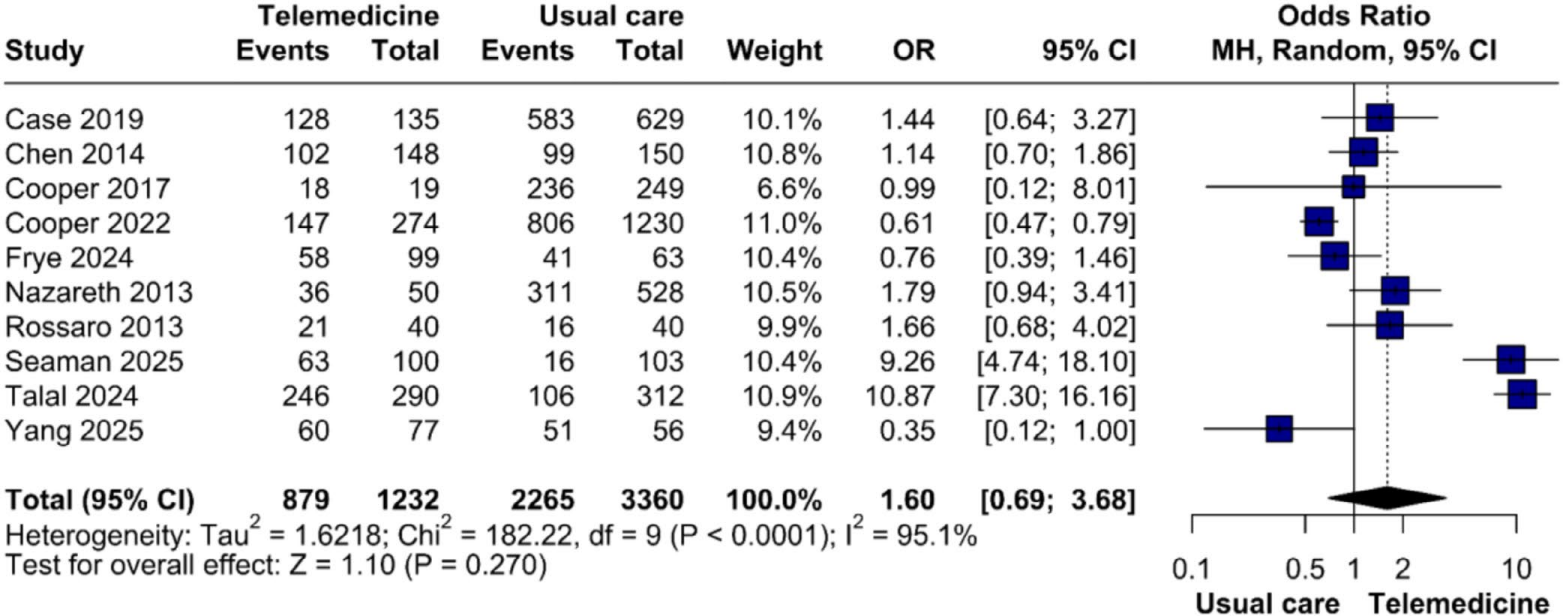
Forest plot of sustained virologic response comparing synchronous telemedicine and in‐person care.

Ten studies compared SVR between TM and in‐person care. The pooled analysis showed no significant difference (OR = 1.60, 95% CI 0.69–3.68; *p* = 0.27), though heterogeneity was substantial (*τ* ^2^ = 1.62, *I* ^2^ = 95.1%) (Figure 1). Sensitivity analysis using the leave‐one‐out method confirmed the lack of statistical significance, with heterogeneity remaining high across all iterations (Figure S4).

### 3.4 Funnel Plot Analysis of SVR

The funnel plot for sustained virologic response is available in Figure S5. Visual inspection reveals some asymmetry, with the two randomised trials showing a larger effect size for telemedicine compared to the observational studies. Despite this, formal statistical testing did not find evidence of significant publication bias (Egger's test: *t* = 0.39, *p* = 0.70; Begg's test: *z* = −0.27, *p* = 0.79). The plot does not show a classic small‐study effect; rather, the asymmetry is likely attributable to the substantial methodological heterogeneity between different study designs.

#### 3.4.1 Secondary Outcomes: Treatment Initiation and Completion

Treatment initiation was reported in five studies. The pooled effect did not reach statistical significance (OR = 7.59, 95% CI 0.79–72.81; *p* = 0.079), and heterogeneity was considerable (*τ* ^2^ = 6.45, *I* ^2^ = 98.2%) (Figure S6). However, exclusion of the study by Lepage et al. [21] yielded a significant benefit for TM (OR = 19.22, 95% CI 11.09–33.32; *p* < 0.0001), with moderate heterogeneity (*τ* ^2^ = 0.1274, *I* ^2^ = 40.8%) (Figure S7).

Treatment completion was assessed in eight studies. Overall, no significant difference was observed (OR = 2.50, 95% CI 0.76–8.25; *p* = 0.134), and heterogeneity was high (*τ* ^2^ = 2.76, *I* ^2^ = 95.0%) (Figure S8). Sensitivity analysis indicated that excluding Cooper et al. [22] favoured TM, although heterogeneity remained substantial (*τ* ^2^ = 1.57, *I* ^2^ = 89.3%) (Figure S9).

### 3.5 Subgroup Analyses—SVR

#### 3.5.1 Rural vs. Non‐Rural Settings

**FIGURE 2:**
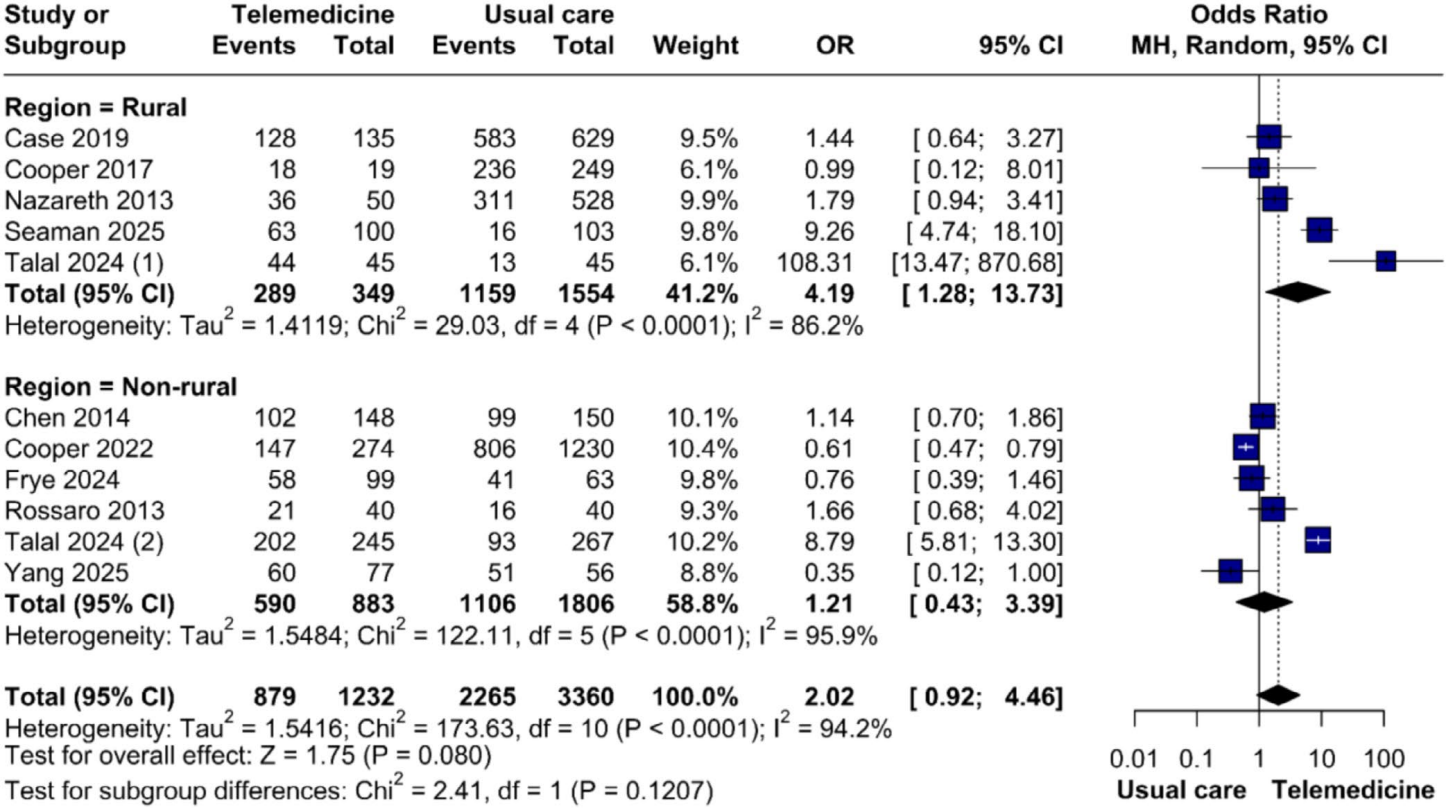
Subgroup analysis of sustained virologic response in rural settings compared with non‐rural settings.

Talal et al. [23] stratified their population by rural and non‐rural areas, so we presented these groups where rurality was a relevant factor. Rural studies [20, 27, 28, 29, 31] demonstrated a significant advantage for TM (OR = 4.19, 95% CI 1.28–13.73; *p* = 0.018), with high heterogeneity (*τ* ^2^ = 1.04, *I* ^2^ = 86.2%). In non‐rural settings [22, 23, 24, 26, 29, 35], no difference was found (OR = 1.21, 95% CI 0.43–3.39; *p* = 0.721), with high heterogeneity (*τ* ^2^ = 1.55, *I* ^2^ = 95.9%) (Figure 2). Leave‐one‐out analysis revealed that the significance in rural settings was largely driven by the studies of Talal et al. [29] and Seaman et al. [31], as their exclusion rendered the results non‐significant (Figure S10).

#### 3.5.2 DAA‐Only Therapies

Six studies [20, 22, 23, 24, 29, 31] evaluated TM in the context of DAAs. No significant difference was found (OR = 1.65, 95% CI 0.41–6.61; *p* = 0.479), with very high heterogeneity (*τ* ^2^ = 2.76, *I* ^2^ = 97.2%) (Figure S11). Sensitivity analyses confirmed robustness, with no meaningful changes (Figure S12).

#### 3.5.3 Study Design (RCTs vs. Observational Studies)

**FIGURE 3:**
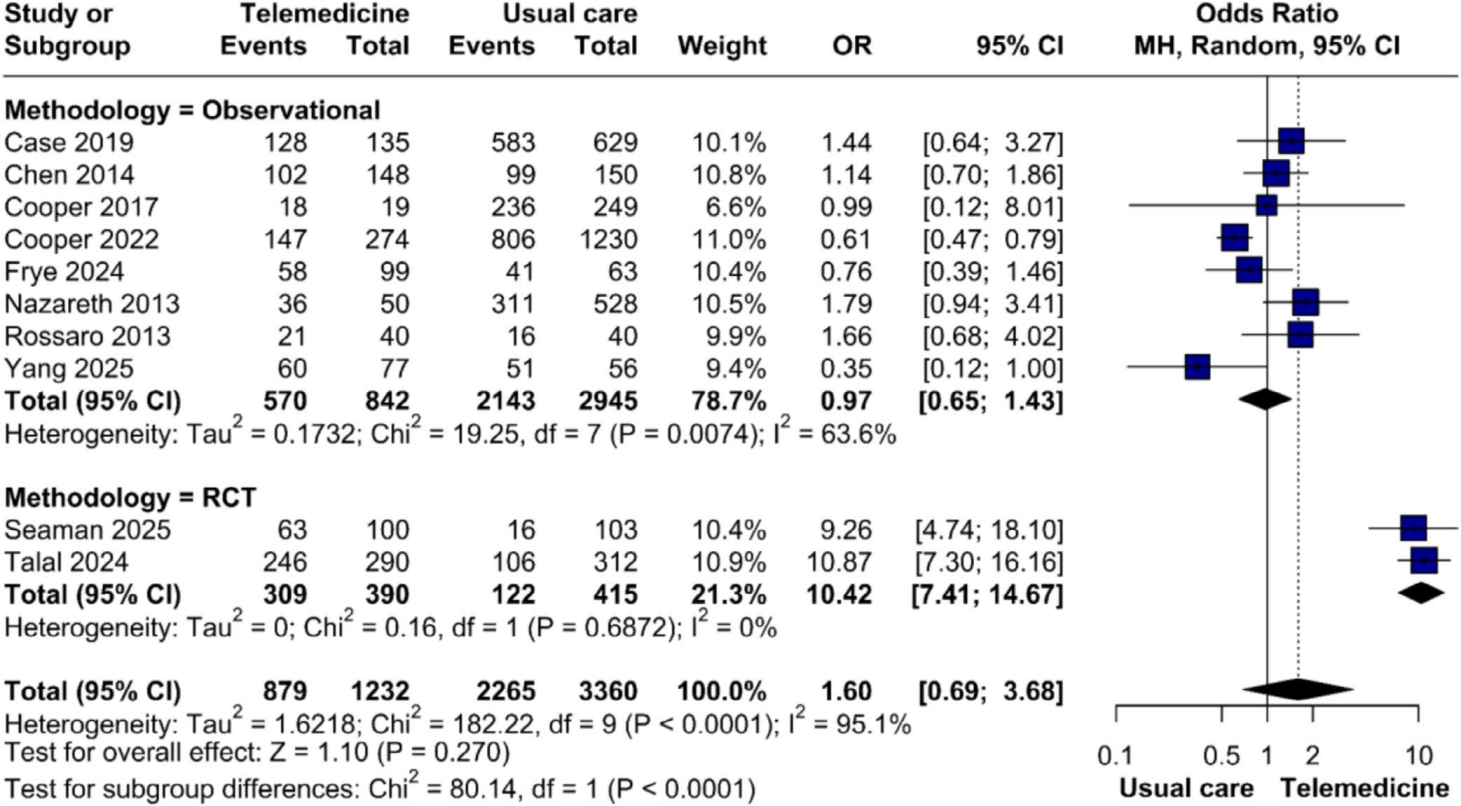
Subgroup analysis of sustained virologic response in randomised controlled trials compared with observational studies.

Eight observational studies [20, 22, 23, 24, 26, 27, 28, 35] showed no difference between TM and in‐person care (OR = 0.97, 95% CI 0.65–1.43; *p* = 0.875), with moderate heterogeneity (*τ* ^2^ = 0.17, *I* ^2^ = 63.6%). By contrast, the two RCTs [29, 31] consistently favoured TM (OR = 10.42, 95% CI 7.41–14.67; *p* < 0.001), with no heterogeneity (*τ* ^2^ = 0, *I* ^2^ = 0%) (Figure 3). These findings were stable on sensitivity analysis for the meta‐analytic results of observational studies (Figure S13).

#### 3.5.4 DAA‐Only Therapies in Rural vs. Non‐Rural Settings

**FIGURE 4:**
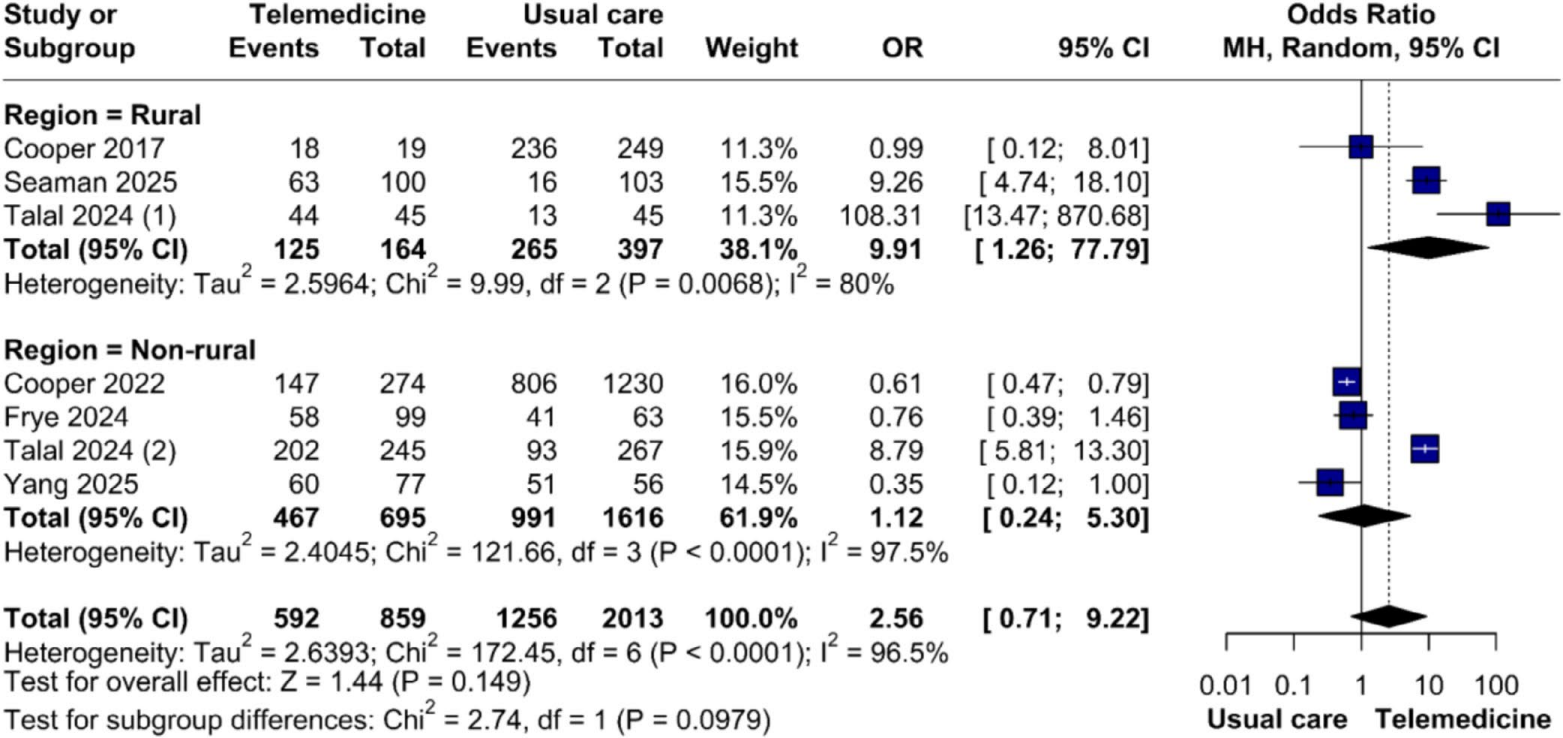
Subgroup analysis of sustained virologic response in studies of direct‐acting antiviral therapy in rural settings compared with non‐rural settings.

Three rural studies [20, 29, 31] showed a strong benefit for TM (OR = 9.91, 95% CI 1.26–77.79; *p* = 0.029), with moderate heterogeneity (*τ* ^2^ = 2.59, *I* ^2^ = 80.0%). Meanwhile, non‐rural studies [22, 23, 24, 29] favoured in‐person care (OR = 1.12, 95% CI 0.24–5.30; *p* = 0.886), with high heterogeneity (*τ* ^2^ = 2.404, *I* ^2^ = 97.5%) (Figure 4).

### 3.6 Subgroup Analyses—Treatment Initiation

#### 3.6.1 Rural vs. Non‐Rural Settings

Two rural studies [21, 31] reported treatment initiation, with no significant difference between groups (OR = 3.37, 95% CI 0.03–433.88; *p* = 0.624), though heterogeneity was very high (*τ* ^2^ = 7.83, *I* ^2^ = 99.0%). In non‐rural settings [32, 33], two studies significantly favoured TM (OR = 11.54, 95% CI 4.97–26.75; *p* < 0.001), with no heterogeneity (*τ* ^2^ = 0, *I* ^2^ = 0%) (Figure S14).

#### 3.6.2 DAA‐Only Therapies

**FIGURE 5:**
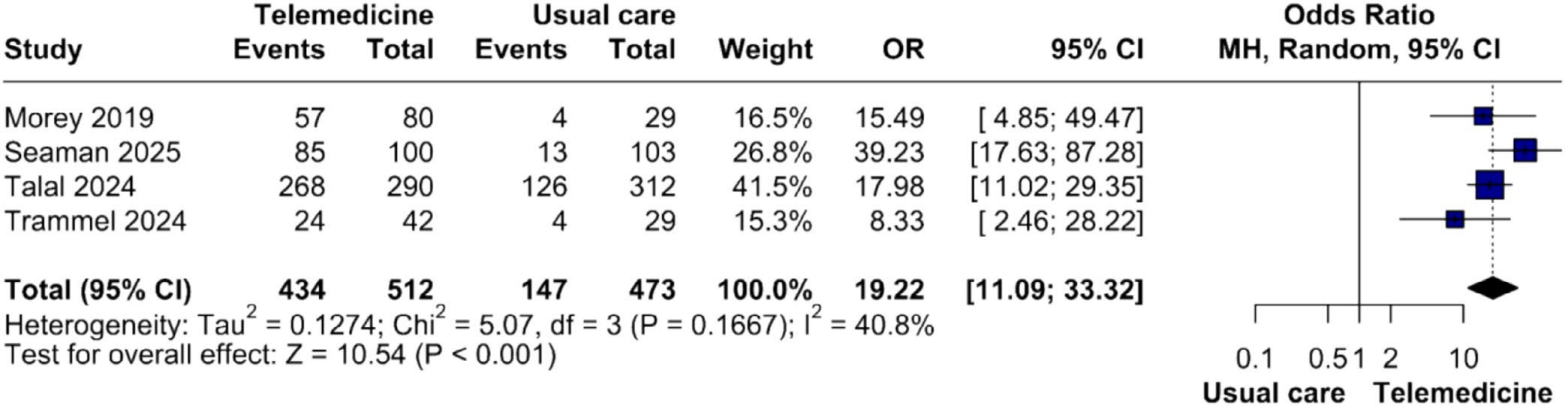
Forest plot of treatment initiation comparing synchronous telemedicine and in‐person care.

Four studies [29, 31, 32, 33] assessing DAAs showed a significant benefit for TM (OR = 19.22, 95% CI 11.09–33.32; *p* < 0.001), with moderate heterogeneity (*τ* ^2^ = 0.13, *I* ^2^ = 40.8%) (Figure 5).

#### 3.6.3 Study Design (RCTs vs. Observational Studies)

Both RCTs [29, 31] favoured TM (OR = 24.86, 95% CI 11.68–52.92; *p* < 0.001), with moderate heterogeneity (*τ* ^2^ = 0.19, *I* ^2^ = 62.6%). In contrast, three observational studies [21, 32, 33] showed no significant difference (OR = 3.24, 95% CI 0.18–59.32; *p* = 0.428), with high heterogeneity (*τ* ^2^ = 6.34, *I* ^2^ = 96.7%) (Figure S15).

#### 3.6.4 DAA‐Only Therapies in Rural vs. Non‐Rural Settings

Only non‐rural settings had a sufficient number of studies for assessment. In this subgroup, two studies [32, 33] again favoured TM (OR = 11.54, 95% CI 4.97–26.75; *p* < 0.001), with no heterogeneity (*τ* ^2^ = 0, *I* ^2^ = 0%) (Figure S16).

### 3.7 Subgroup Analyses—Treatment Completion

#### 3.7.1 Rural vs. Non‐Rural Settings

Two rural studies [27, 31] reported completion rates, showing no significant benefit for TM (OR = 3.13, 95% CI 0.36–27.04; *p* = 0.299), with high heterogeneity (*τ* ^2^ = 2.137, *I* ^2^ = 88.1%). In non‐rural settings, five studies [22, 23, 24, 26, 30] found no significant difference (OR = 1.51, 95% CI 0.54–4.22; *p* = 0.429), again with high heterogeneity (*τ* ^2^ = 1.15, *I* ^2^ = 86.2%) (Figure S17). These results were stable when leave‐one‐out analysis was applied in studies in non‐rural settings (Figure S18).

#### 3.7.2 DAA‐Only Therapies

Six studies [22, 23, 24, 29, 30, 31] focusing on DAAs showed no significant difference overall (OR = 2.78, 95% CI 0.63–12.28; *p* = 0.177), with very high heterogeneity (*τ* ^2^ = 3.26, *I* ^2^ = 96.4%) (Figure S19). Notably, removing Cooper et al. [22] yielded a significant advantage for TM (OR = 4.10, 95% CI 1.21–13.9; *p* = 0.0236), though heterogeneity remained high (*τ* ^2^ = 1.73, *I* ^2^ = 91.3%) (Figure S20).

#### 3.7.3 Study Design (RCTs vs. Observational Studies)

Two RCTs [29, 31] favoured TM (OR = 12.89, 95% CI 7.93–20.94; *p* < 0.001), with low heterogeneity (*τ* ^2^ = 0.04, *I* ^2^ = 25.5%). Six observational studies [22, 23, 24, 26, 27, 30] showed no difference (OR = 1.41, 95% CI 0.58–3.41; *p* = 0.446), with high heterogeneity (*τ* ^2^ = 0.97, *I* ^2^ = 82.8%) (Figure S21). Sensitivity analysis confirmed stability of these findings when including only observational studies (Figure S22).

#### 3.7.4 DAA‐Only Therapies in Rural vs. Non‐Rural Settings

Only non‐rural settings had a sufficient number of studies for assessment. Among four non‐rural studies [22, 23, 24, 26, 30], no significant difference was observed (OR = 1.27, 95% CI 0.41–3.94; *p* = 0.684), with high heterogeneity (*τ* ^2^ = 1.13, *I* ^2^ = 86.2%) (Figure S23).

### 3.8 Systematic Review

#### 3.8.1 Telemedicine for PWUD With Hepatitis C

In a prospective cohort study in Gran Canaria, Spain, Morales‐Arraez et al. [30] evaluated a TM‐based model for re‐engaging PWUD with HCV who were lost to follow‐up, comparing it to standard hospital‐based care. Patients at two drug treatment centers in Tenerife were offered either TM integrated with decentralised HCV treatment at the centers (*n* = 32) or standard referral to hospital specialists (*n* = 37). TM patients were more likely to have a history of injection or inhaled drug use (100% vs. 86.5%; *p* = 0.057) and to be on opioid substitution therapy (100% vs. 69.2%; *p* = 0.010). Re‐engagement program completion was higher with TM (62.5% [95% CI: 45.8–79.2]) compared to standard care (24.3% [95% CI: 10.5–38.1]; *p* = 0.002), and among prior non‐attenders (64.7% vs. 25.9%; *p* = 0.015). SVR was achieved in all who completed treatment, with SVR assessed in 75% of TM and 70% of standard care patients.

In a single‐center retrospective cohort study of pregnant PWUD [32], individuals with active HCV were divided into pre‐TM (*n* = 29) and post‐TM (*n* = 42) groups following the implementation of remote infectious disease consultations during the COVID‐19 pandemic. After TM became available, HCV‐positive patients were significantly more likely to attend their ID consultation (44% [95% CI: 29.1–57.8] vs. 19% [95% CI: 9.8–38.4]; *p* = 0.03) and to initiate HCV treatment (57% [95% CI: 42.2–70.9] vs. 14% [95% CI: 5.5–30.6]; *p* < 0.01) compared to the pre‐TM cohort.

The Peer TeleHCV trial [31] was an open‐label randomised controlled trial conducted across seven rural Oregon counties, targeting PWUD with high rates of HCV infection and opioid use. A total of 203 participants were randomised to peer‐assisted TM (*n* = 100) or usual care (*n* = 103). Treatment initiation was substantially higher in the TeleHCV arm (85% vs. 13%) (RR 6.73, 95% CI: 4.02–11.30; *p* < 0.001), and treatment completion was also higher (46% vs. 9%) (RR 5.30, 95% CI: 2.70–10.20; *p* < 0.001). SVR at 12 weeks post‐treatment or mock completion was achieved by 63% of TeleHCV participants versus 16% of usual care (RR 4.06, 95% CI: 2.52–6.52; *p* < 0.001). Among those initiating treatment, SVR12 rates were comparable (74% vs. 77%).

In a multisite, pragmatic stepped‐wedge cluster randomised trial [29] conducted across 12 opioid treatment programs in New York State between 2017 and 2020, 602 HCV‐seropositive individuals receiving opioid use disorder care were randomised to usual referral to an off‐site hepatitis specialist (*n* = 312) or facilitated TM (*n* = 290). The primary outcome was SVR 12 weeks after DAA therapy completion. Intention‐to‐treat analysis showed significantly higher SVR rates in the TM group compared with referral (90.3% vs. 39.4%). Among participants who initiated therapy, observed SVR was also higher in the TM group (91.8% vs. 84.1%). TM accelerated care engagement, with shorter time from initial visit to DAA initiation (mean 49.9 vs. 123.5 days; *p* < 0.001), and achieved substantially higher treatment initiation rates (92.4% vs. 40.4%). Adherence was very high in both groups, and reinfection rates during follow‐up were similar.

### 3.9 Synchronous Telemedicine for Incarcerated Populations With HCV

In a quasi‐experimental before‐and‐after observational study with prospective data collection conducted in the prison system of North East England, Morey et al. [33] evaluated the impact of synchronous TM on treatment outcomes for incarcerated individuals with HCV, with a particular focus on HMP Northumberland. 80 inmates with confirmed HCV infection were assessed through a TM clinic led by a hepatologist and supported by an in‐reach nursing team, replacing the need for off‐site hospital visits. Of these, 57 (71%) initiated DAA therapy, and 42 (≈73%) completed treatment within the prison, while the remainder were released or transferred during therapy. Attendance rates were high (83%), patient satisfaction favourable (80% rated the service as “good” or “excellent”), and among those with available outcomes (*n* = 29), SVR was 100%. Notably, in the year preceding TM implementation, only four inmates had initiated treatment, highlighting a > 10‐fold increase in therapy initiation following program adoption.

### 3.10 Pandemic‐Driven Shifts to Telemedicine in HCV Care

In a retrospective chart review conducted at the Grady Liver Clinic in Atlanta, USA, Frye et al. [23] evaluated the effectiveness of HCV treatment delivered through TM compared with the traditional in‐person model. The study included 640 patients, with 388 enrolled in the pre‐pandemic period (March 2019–February 2020), when care was exclusively in person, and 252 during the pandemic period (March 2020–February 2021), when in‐person, TM, and hybrid modalities were introduced. The pandemic period was associated with longer waiting times to initiate therapy, yet cure rates (SVR12) remained high and statistically comparable between cohorts (ITT: 95.8% vs. 95.1%) and across care modalities. The principal losses occurred in post‐treatment follow‐up, as approximately 25%–30% of patients did not undergo SVR12 testing, while treatment discontinuation was low in both periods. Overall, the transition to TM preserved the effectiveness of HCV therapy in a medically underserved population.

In a retrospective cohort study conducted at the specialty pharmacy and infusion services of UK HealthCare in Lexington, Kentucky, Cooper et al. [22] evaluated the impact of the COVID‐19 pandemic and the transition to a TM model on SVR data collection and other HCV treatment outcomes. A total of 1504 patients were included: 1230 in the pre‐COVID‐19 cohort (January 2018–March 2020) and 274 in the COVID‐19 cohort (March–November 2020). In the ITT analysis, confirmed SVR12 was reduced during the pandemic (53.6% vs. 65.5%; *p* < 0.001). However, among patients who completed therapy and obtained follow‐up labs, SVR12 rates were nearly identical (97.9% vs. 98.0%). These findings indicate that while DAA efficacy was preserved, the pandemic‐driven transition to telehealth introduced systemic barriers that reduced adherence and follow‐up rather than therapeutic effectiveness.

### 3.11 Economic Aspects of Telemedicine for HCV

In a prospective cohort study conducted across 12 hospitals in Taiwan, Chen et al. [35] compared the economic and clinical outcomes of a nurse‐led outpatient support program versus a telecare intervention for 298 patients with chronic HCV treated with peginterferon alfa‐2a plus ribavirin. Patients were randomised into two groups: conventional in‐person nursing support (*n* = 150) and TM consultation via a health communication center (*n* = 148). The primary outcome, SVR, was similar (66.0% vs. 68.9%), but TM significantly reduced treatment discontinuation (12.0% vs. 5.4%; *p* < 0.05), reflecting improved adherence (88.0% vs. 94.6%). Program costs were nearly halved in the TM group (US$112,500 vs. US$232,632), representing a 51.6% reduction in expenditures.

In an observational cost‐minimisation study conducted at El Dueso prison in Spain, Cuadrado et al. [34] compared TM with usual care for inmates receiving DAAs for HCV. Seventy‐five patients under the TM model were evaluated, and outcomes were assumed equivalent to usual care, allowing for a pure economic comparison. During one‐year follow‐up, the mean consultation cost per patient was higher with TM (€752.7 vs. €666.2), but overall cohort costs were lower (€87,861 vs. €126,537), representing a total saving of €38,677 (30.6%). The mean cost per patient was €1171 with TM versus €1687 with usual care, with average savings of €516 per patient. Importantly, 99.3% of savings were attributed to reduced prison‐to‐hospital transfers. Clinical effectiveness was preserved (SVR 94.7% after first‐line, 100% after retreatment), and patient satisfaction was very high (median 5/5 across survey items). TM was more efficient and acceptable than traditional care in penitentiary settings, primarily by reducing logistical and transfer‐related costs while maintaining effectiveness and satisfaction.

## 4 Discussion

### 4.1 Summary of Findings

This systematic review and meta‐analysis found that synchronous TM achieves outcomes broadly comparable to in‐person care across SVR, treatment initiation, and treatment completion. In the pooled analyses, no significant differences were observed for the primary or secondary outcomes, though high between‐study heterogeneity was frequently present. Sensitivity analyses using the leave‐one‐out method generally confirmed the stability of the pooled estimates, indicating that most results were not driven by any single study. However, in some secondary analyses—particularly for treatment initiation and completion—the exclusion of specific studies (e.g., Lepage et al. or Cooper et al.) revealed statistically significant benefits favoring TM, underscoring that some findings remain sensitive to individual study effects.

Subgroup analyses provided further nuance. In rural settings and RCTs, TM consistently demonstrated significant advantages, particularly for treatment initiation and completion, with sensitivity analyses confirming robustness in these subgroups. In contrast, observational studies and analyses conducted in non‐rural settings generally showed equivalence, with some evidence suggesting that in DAA‐only non‐rural cohorts, in‐person care performed better. Importantly, sensitivity analyses confirmed these patterns, highlighting that the rural and RCT findings were stable, whereas certain observational comparisons remained less reliable due to residual heterogeneity.

The systematic review component reinforced these quantitative findings by contextualising TM's role among key populations. In PWUD, models integrating peer support or embedding TM within opioid treatment programs dramatically increased linkage to care, treatment initiation, and cure rates compared to referral‐based approaches. Among incarcerated individuals, TM overcame logistical and security barriers, leading to a > 10‐fold increase in treatment uptake with excellent virological outcomes. During the COVID‐19 pandemic, TM preserved antiviral efficacy but introduced barriers to adherence and SVR ascertainment, primarily due to reduced follow‐up testing. Finally, economic evaluations consistently indicated that TM reduces healthcare costs—especially those related to transportation and hospital transfers—while maintaining or improving clinical effectiveness.

Taken together, the evidence suggests that synchronous TM achieves outcomes comparable to in‐person care and may be superior in specific contexts, particularly in rural and marginalised populations. While most findings were robust to sensitivity analyses, certain subgroup effects were sensitive to the inclusion of individual studies, emphasising the need for cautious interpretation and the value of high‐quality RCTs to confirm these signals.

### 4.2 Comparison With Existing Literature

While building upon prior work such as the review by Haridy et al. [7], our study provides a more focused and in‐depth analysis. We specifically concentrated on synchronous TM for the management of HCV. By including a larger, more contemporary cohort of studies, our analysis was expanded beyond the primary outcome of SVR to also include treatment initiation and completion. Crucially, we conducted extensive subgroup analyses that were essential for interpreting the high observed heterogeneity, and we complemented our quantitative findings with a narrative synthesis on key topics like telemedicine's role for PWUD and incarcerated populations. These results strongly support recommendations from the American Association for the Study of Liver Diseases (AASLD) and the Infectious Diseases Society of America (IDSA) to use TM as a key strategy to overcome geographical barriers to specialist care, especially in rural areas [36].

In a large umbrella review of 83 systematic reviews, Snoswell et al. [37] reported that telehealth interventions were generally equivalent or superior to conventional care across multiple clinical domains, with no evidence of harm. This reinforces our observation that synchronous TM for HCV achieves outcomes at least comparable to in‐person care and, in certain contexts, may offer significant advantages. Notably, Snoswell and colleagues emphasised that telehealth benefits were particularly evident when interventions addressed access barriers and were tailored to the needs of specific populations—an observation that parallels our subgroup findings.

### 4.3 Limitations

This review has several limitations. Most included studies were observational and at serious risk of bias, particularly from confounding and participant selection, likely diluting the apparent effect of TM. High statistical heterogeneity persisted even after subgrouping, reflecting variation in interventions, populations, and health systems. Our findings also rest on only two RCTs, which, although rigorous and consistent in showing a strong benefit of TM, limit the generalisability of trial‐level evidence. Sensitivity analyses further highlighted fragility in some results: in several secondary outcomes (e.g., treatment initiation, completion), removing a single influential study changed findings from nonsignificant to statistically significant, indicating that certain effects are sensitive to individual study weight rather than broadly consistent across the evidence base. Pandemic‐era studies also introduced challenges, as reduced follow‐up may have underestimated SVR.

Of note, subgroup analysis of DAA‐only therapies suggested a geographic divergence: TM appeared highly effective in rural contexts but, paradoxically, in‐person care sometimes performed better in non‐rural settings. This finding is not definitive but highlights that local barriers and resources likely shape the comparative effectiveness of care models and underscores the importance of context in interpreting telemedicine outcomes.

### 4.4 Interpretation of Heterogeneity and Bias

The substantial methodological heterogeneity between RCTs and observational studies warrants careful interpretation, particularly regarding the direction of bias. In real‐world observational settings, allocation to telemedicine is rarely random; rather, it is frequently subject to confounding by indication. Telemedicine is often deployed specifically for “hard‐to‐reach” populations—such as individuals in rural/remote areas, those with unstable housing, or people who use drugs—who face structural barriers that make in‐person attendance difficult or impossible. Conversely, patients retained in standard in‐person care often represent a more stable population with fewer social determinants of health negatively impacting their prognosis. Therefore, the observational telemedicine cohorts likely possessed a higher baseline risk of non‐adherence and loss to follow‐up compared to their in‐person counterparts.

The contrast between RCTs and observational studies in our subgroup analyses supports this interpretation. Both randomised trials were at low risk of bias and consistently demonstrated large and statistically significant benefits of synchronous TM across SVR, treatment initiation, and completion. In contrast, the pooled observational data—with substantial residual confounding—showed no significant difference between TM and in‐person care despite similar treatment regimens and follow‐up procedures. Taken together, these findings suggest that the lack of effect in observational cohorts likely reflects systematic biases inherent to real‐world service allocation rather than a true equivalence or inferiority of telemedicine. Thus, the direction of bias across observational studies appears strongly oriented toward the null, providing a conservative estimate of the impact of telemedicine and underscoring the importance of the randomised evidence supporting its effectiveness.

### 4.5 Contribution to the Literature

This study provides the most detailed synthesis of synchronous TM for HCV to date, extending prior reviews by including initiation and completion outcomes, conducting extensive subgroup analyses, and integrating evidence from key populations and economic evaluations. Importantly, it is the first to explicitly demonstrate the divergence between RCTs, which consistently favour TM, and observational studies, which often show equivalence. By highlighting context‐specific effectiveness, this work advances understanding of where and for whom TM is most impactful and underscores its role in accelerating HCV elimination goals.

## 5 Conclusion

Synchronous telemedicine achieves outcomes comparable to in‐person care for the management of chronic HCV, with evidence suggesting superiority in rural and marginalised populations, particularly when programs are integrated into existing care structures. While high heterogeneity and reliance on observational studies limit certainty, randomised evidence consistently favours TM. By improving access, reducing costs, and addressing inequities in care delivery, synchronous TM represents a scalable strategy to advance HCV elimination goals. Future high‐quality RCTs are essential to refine models, evaluate implementation in non‐rural settings, and assess long‐term outcomes.

## Conflicts of Interest

G.G.L.C. has received a research grant from IPSEN. The other authors have no conflicts of interest to declare.

## Supporting information


**Figure S1:** Preferred Reporting Items for Systematic Reviews and Meta‐Analyses flow diagram of the study selection process.

**Figure S2:** Traffic‐light plot of the risk of bias assessment for randomised controlled trials.

**Figure S3:** Traffic‐light plot of the risk of bias assessment for observational studies.

**Figure S4:** Leave‐one‐out sensitivity analysis for sustained virologic response comparing synchronous telemedicine and in‐person care.

**Figure S5:** Funnel plot of sustained virologic response comparing synchronous telemedicine and in‐person care.

**Figure S6:** Forest plot of treatment initiation comparing synchronous telemedicine and in‐person care.

**Figure S7:** Leave‐one‐out sensitivity analysis for treatment initiation comparing synchronous telemedicine and in‐person care.

**Figure S8:** Forest plot of treatment completion comparing synchronous telemedicine and in‐person care.

**Figure S9:** Leave‐one‐out sensitivity analysis for treatment completion comparing synchronous telemedicine and in‐person care.

**Figure S10:** Leave‐one‐out sensitivity analysis for sustained virologic response in rural settings compared with non‐rural settings.

**Figure S11:** Forest plot of sustained virologic response in studies of direct‐acting antiviral therapy comparing synchronous telemedicine and in‐person care.

**Figure S12:** Leave‐one‐out sensitivity analysis for sustained virologic response in studies of direct‐acting antiviral therapy comparing synchronous telemedicine and in‐person care.

**Figure S13:** Sensitivity analysis for sustained virologic response in observational studies comparing synchronous telemedicine and in‐person care.

**Figure S14:** Subgroup analysis of treatment initiation in rural settings compared with non‐rural settings.

**Figure S15:** Subgroup analysis of treatment initiation in randomised controlled trials compared with observational studies.

**Figure S16:** Subgroup analysis of treatment initiation in studies of direct‐acting antiviral therapy in rural settings compared with non‐rural settings.

**Figure S17:** Subgroup analysis of treatment completion in rural settings compared with non‐rural settings.

**Figure S18:** Leave‐one‐out sensitivity analysis for treatment completion in non‐rural settings.

**Figure S19:** Subgroup analysis of treatment completion in studies of direct‐acting antiviral therapy comparing synchronous telemedicine and in‐person care.

**Figure S20:** Leave‐one‐out sensitivity analysis for treatment completion in studies of direct‐acting antiviral therapy comparing synchronous telemedicine and in‐person care.

**Figure S21:** Subgroup analysis of treatment completion in randomised controlled trials compared with observational studies.

**Figure S22:** Sensitivity analysis for treatment completion in observational studies comparing synchronous telemedicine and in‐person care.

**Figure S23:** Subgroup analysis of treatment completion in studies of direct‐acting antiviral therapy in rural settings compared with non‐rural settings.

**Table S1:** Preferred Reporting Items for Systematic Reviews and Meta‐Analyses 2020 checklist.

**Table S2:** Complete electronic search strategy for all databases.

**Table S3:** Definitions of telemedicine, sustained virologic response, and inclusion criteria across the included studies.

## Data Availability

The data that support the findings of this study are available from the corresponding author upon reasonable request.
