# Supplementary figures and images for: Synchronous Telemedicine Versus In‐Person Care in Hepatitis C Treatment: A Systematic Review and Meta‐Analysis
Source: J Viral Hepat. 2026 Jan 28;33(3):e70144. doi: 10.1111/jvh.70144 (PMC12848981; doi:10.1111/jvh.70144)

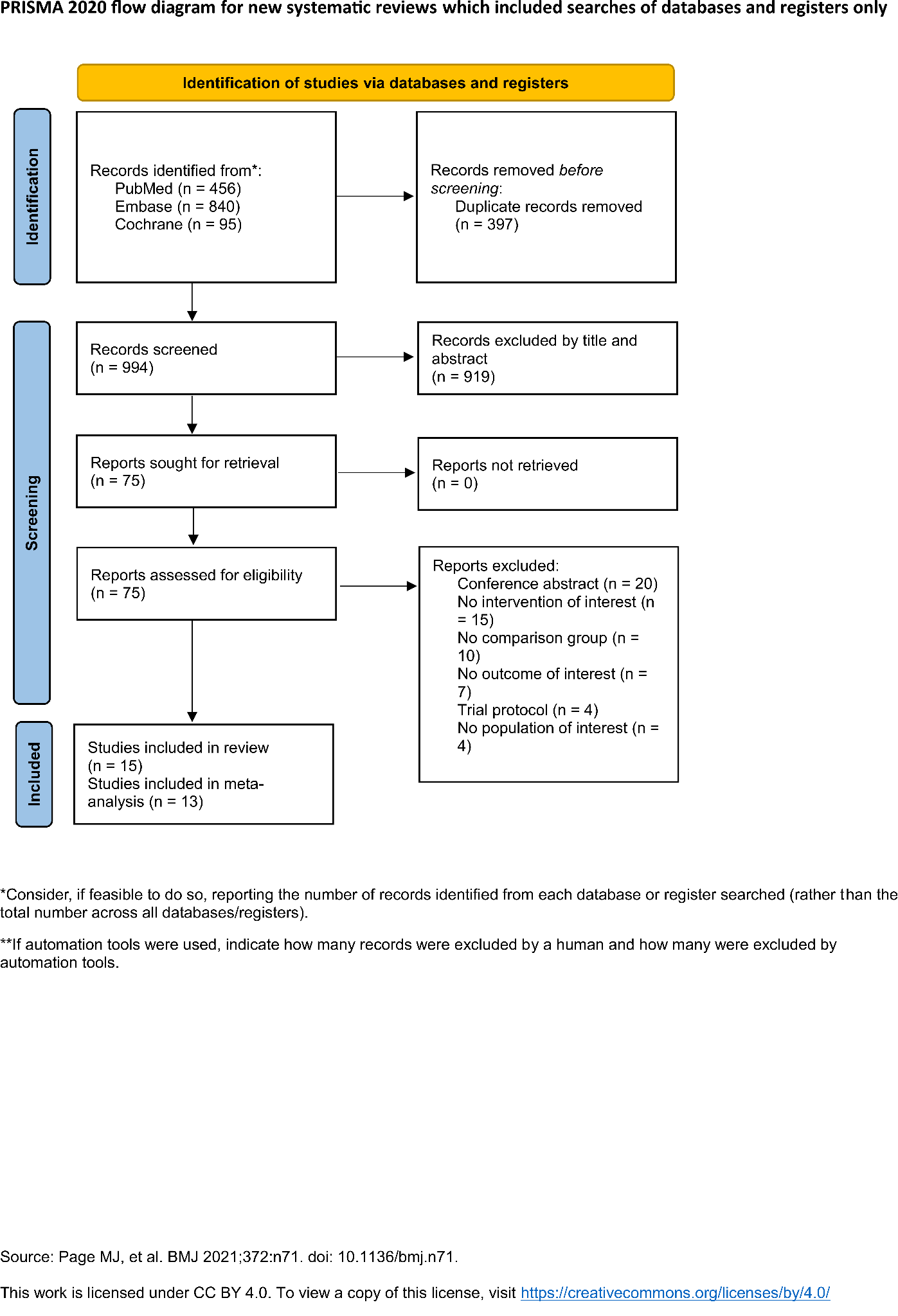

Supplement: Supplementary file 1 — Figure S1: Preferred Reporting Items for Systematic Reviews and Meta‐Analyses flow diagram of the study selection process. [file JVH-33-0-s008.docx]

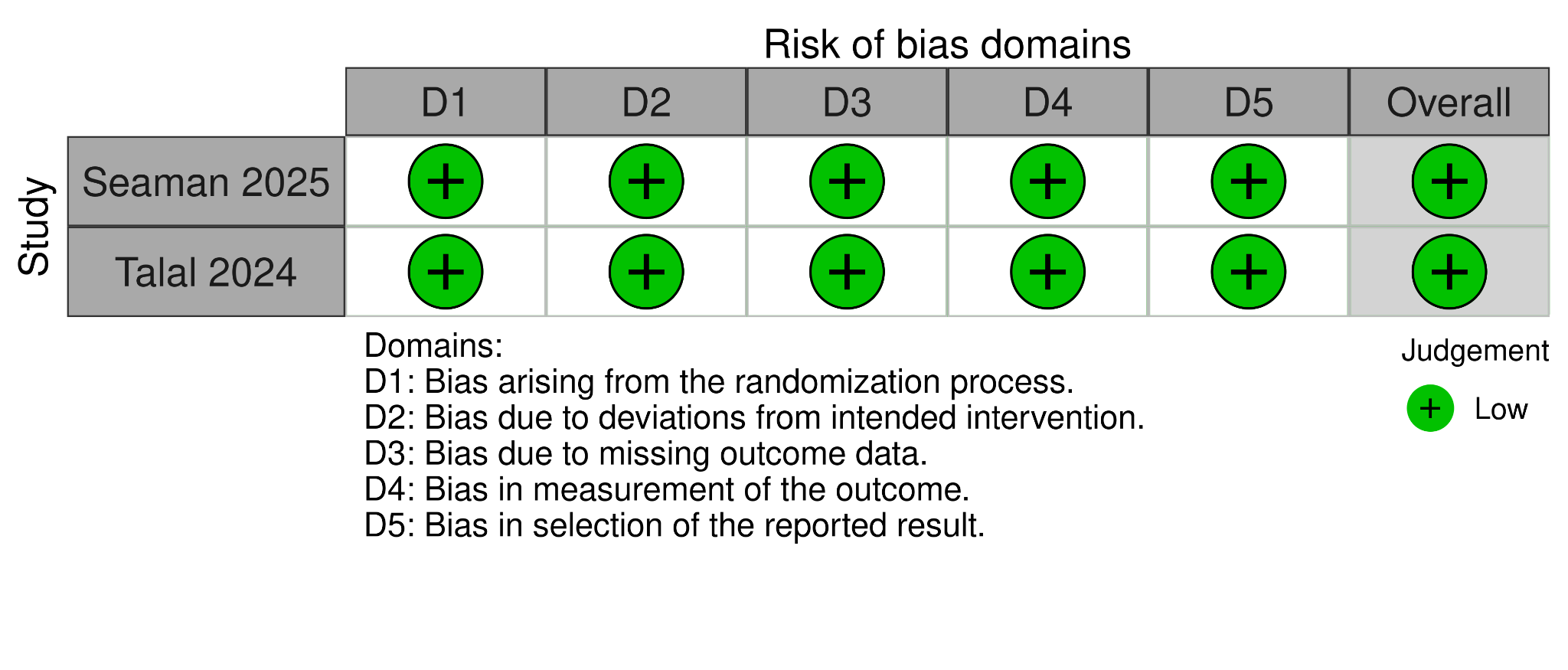

Supplement: Supplementary file 2 — Figure S2: Traffic‐light plot of the risk of bias assessment for randomised controlled trials. [file JVH-33-0-s023.docx]

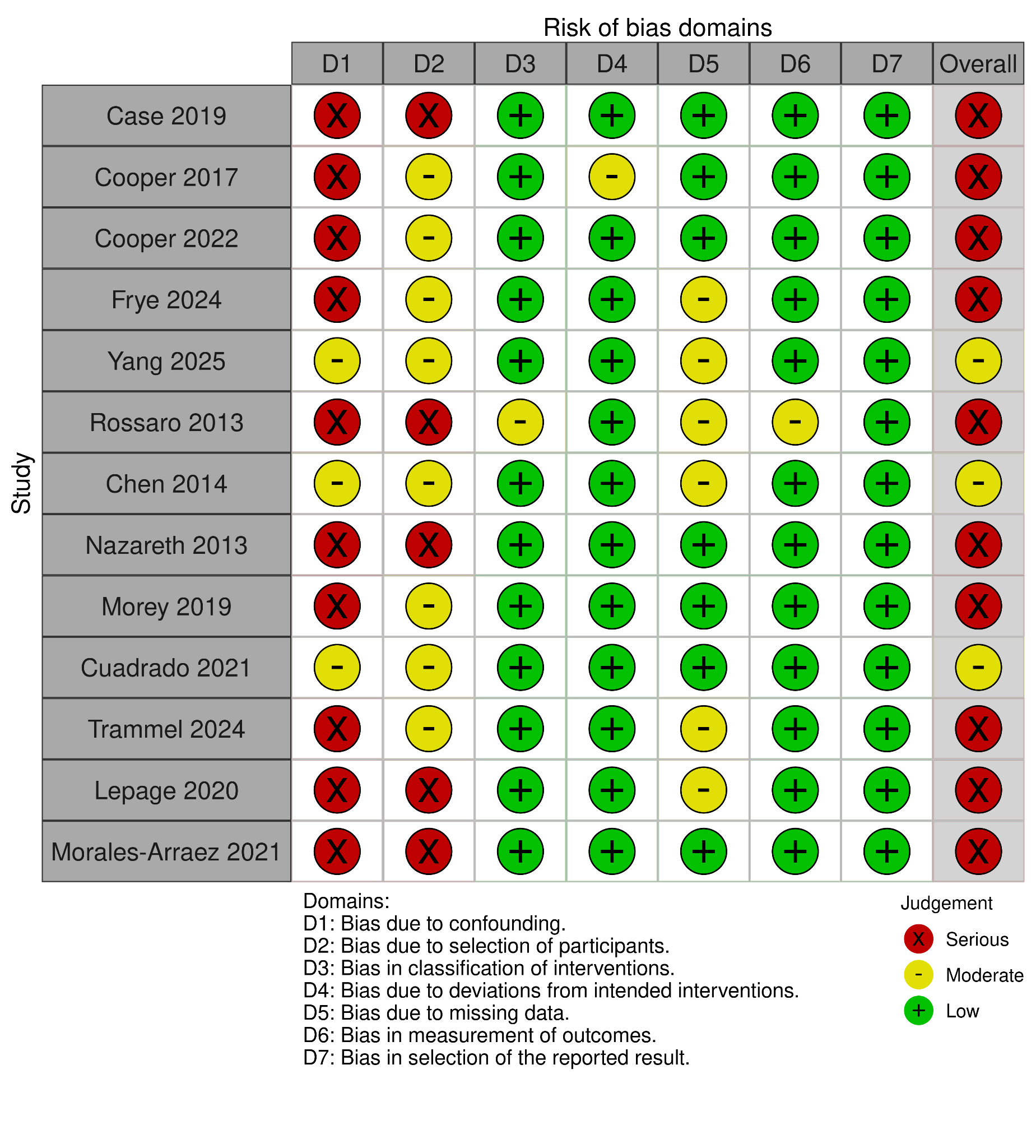

Supplement: Supplementary file 3 — Figure S3: Traffic‐light plot of the risk of bias assessment for observational studies. [file JVH-33-0-s016.docx]

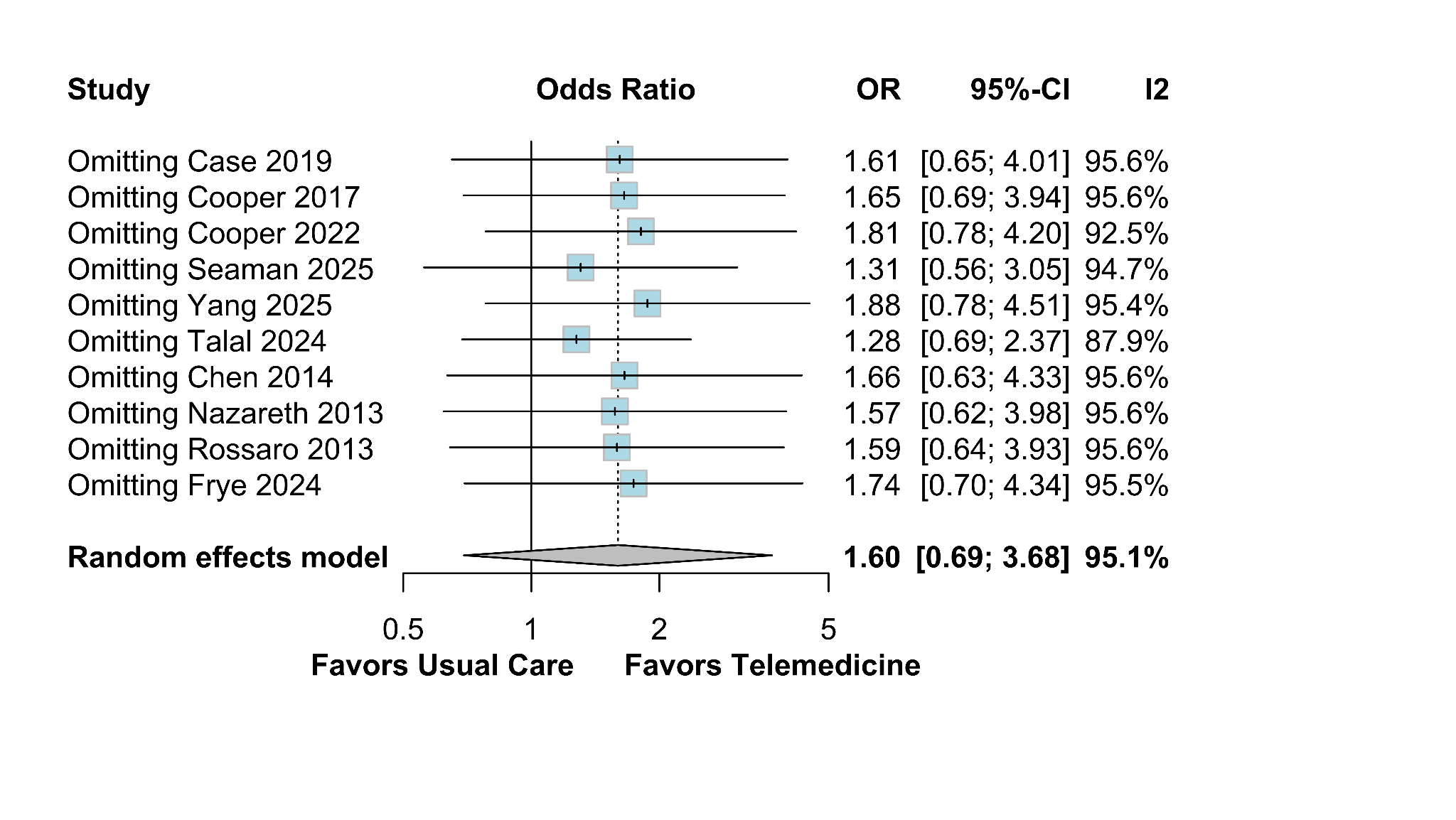

Supplement: Supplementary file 4 — Figure S4: Leave‐one‐out sensitivity analysis for sustained virologic response comparing synchronous telemedicine and in‐person care. [file JVH-33-0-s003.docx]

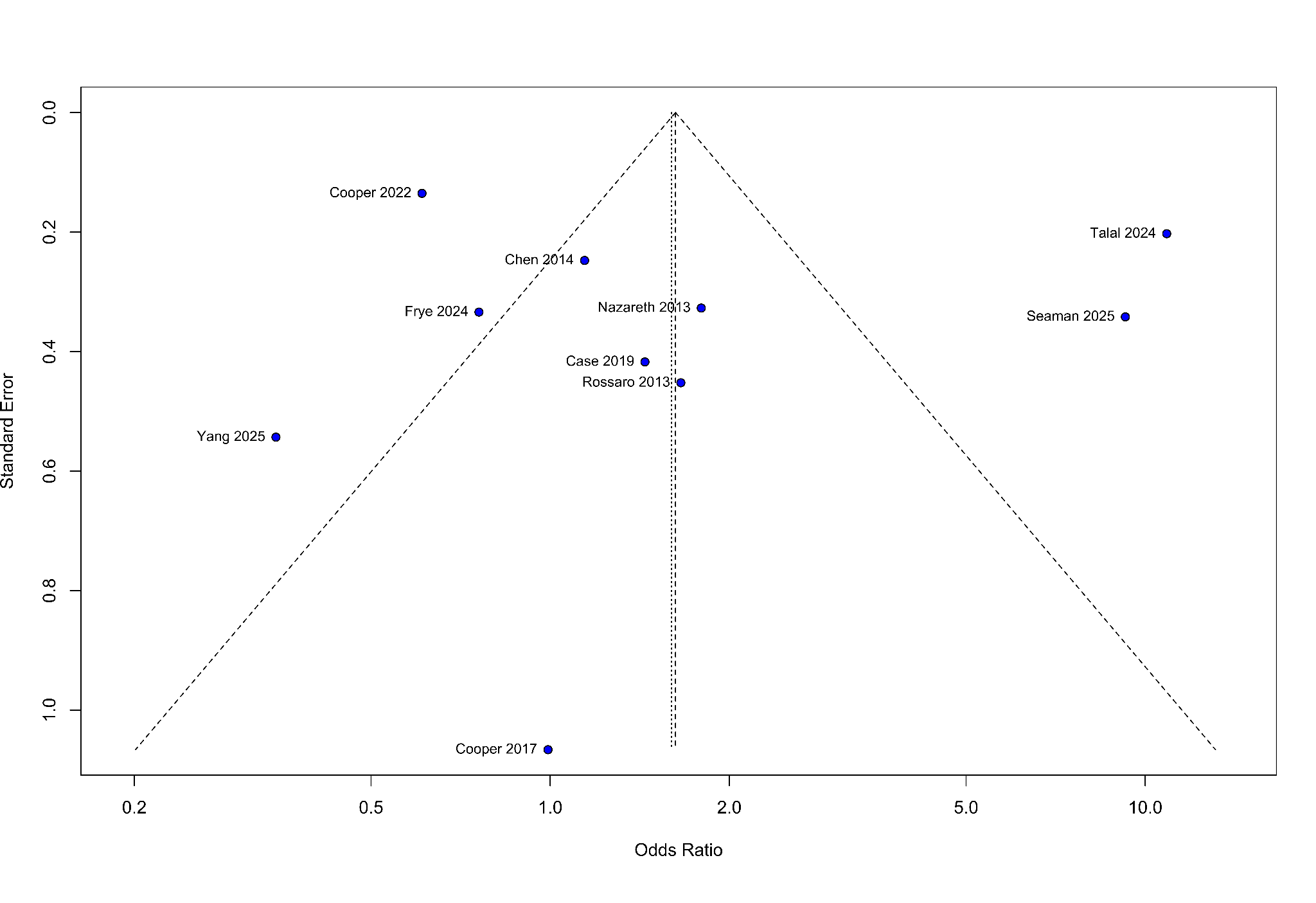

Supplement: Supplementary file 5 — Figure S5: Funnel plot of sustained virologic response comparing synchronous telemedicine and in‐person care. [file JVH-33-0-s011.docx]

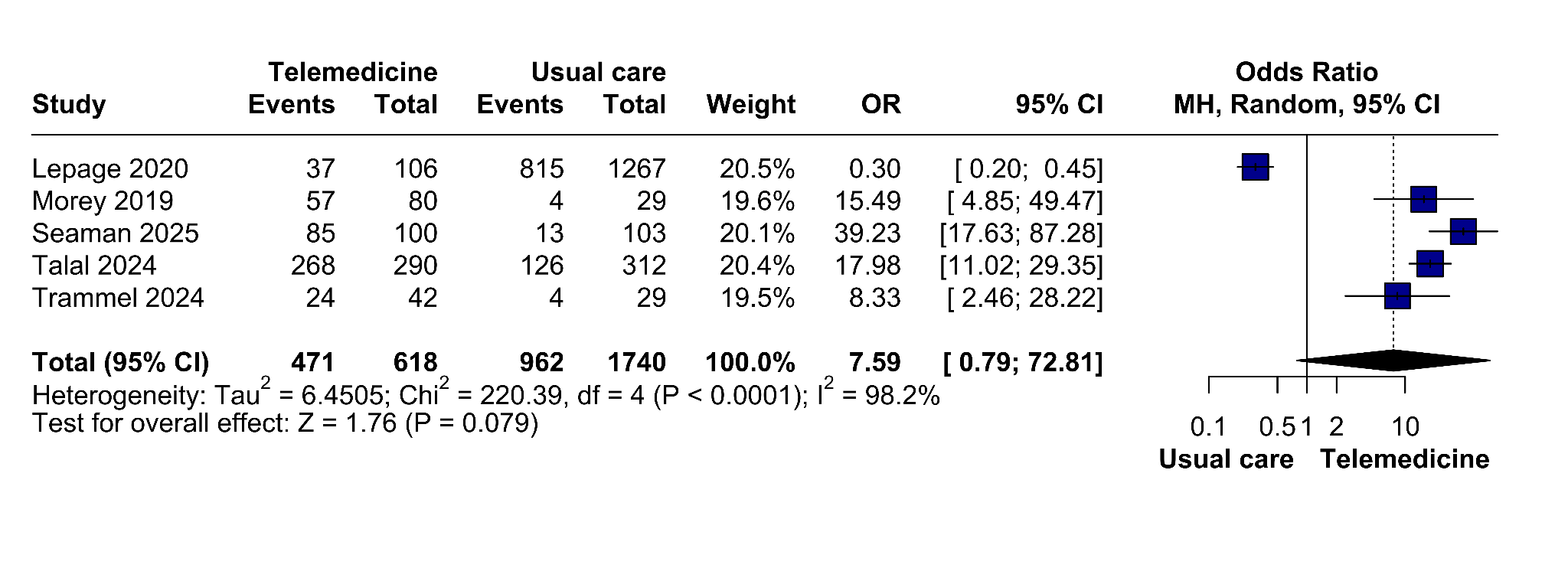

Supplement: Supplementary file 6 — Figure S6: Forest plot of treatment initiation comparing synchronous telemedicine and in‐person care. [file JVH-33-0-s020.docx]

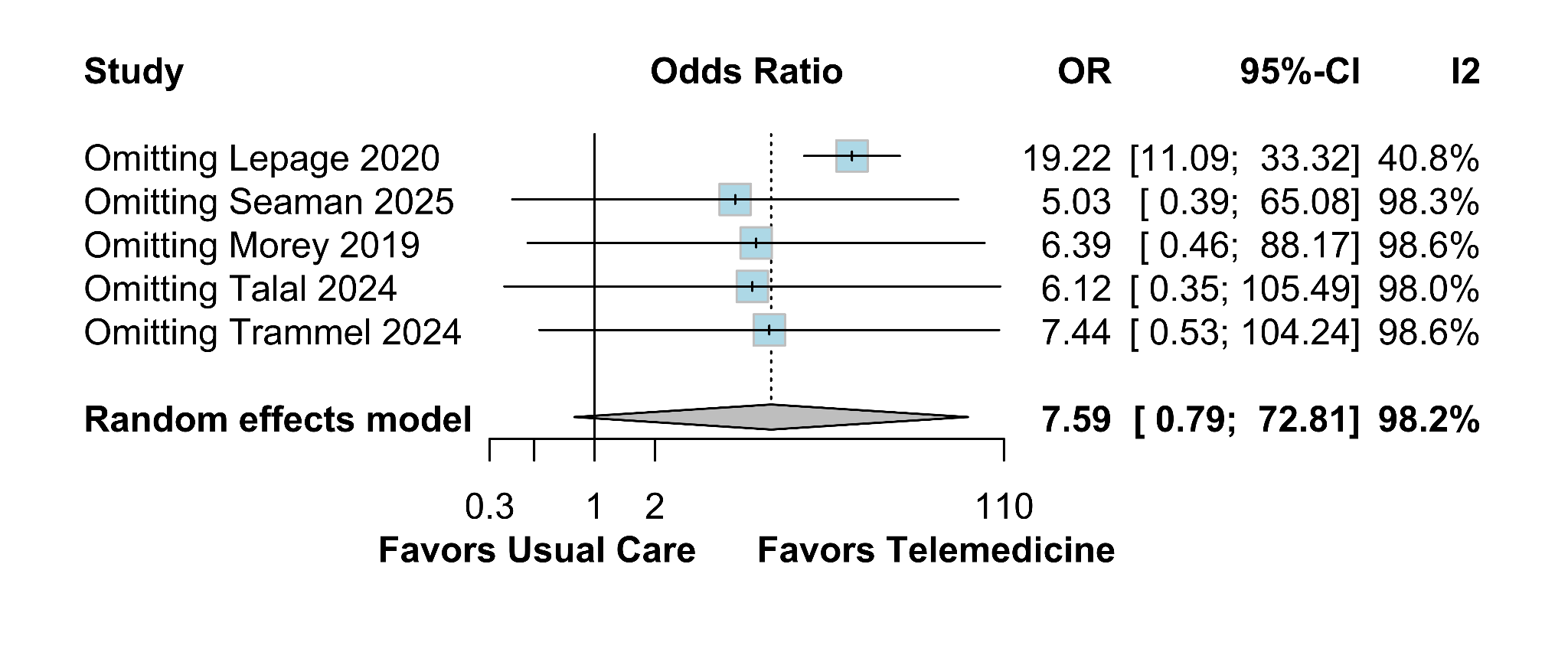

Supplement: Supplementary file 7 — Figure S7: Leave‐one‐out sensitivity analysis for treatment initiation comparing synchronous telemedicine and in‐person care. [file JVH-33-0-s019.docx]

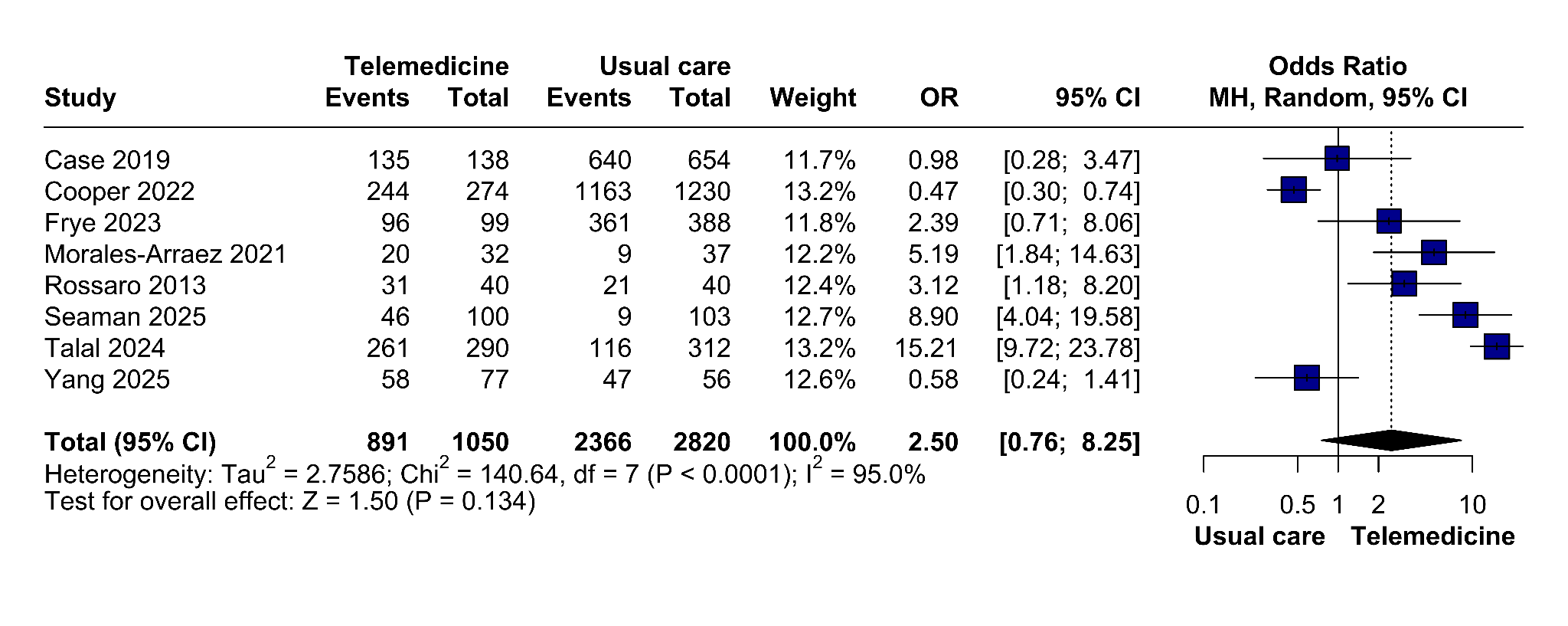

Supplement: Supplementary file 8 — Figure S8: Forest plot of treatment completion comparing synchronous telemedicine and in‐person care. [file JVH-33-0-s002.docx]

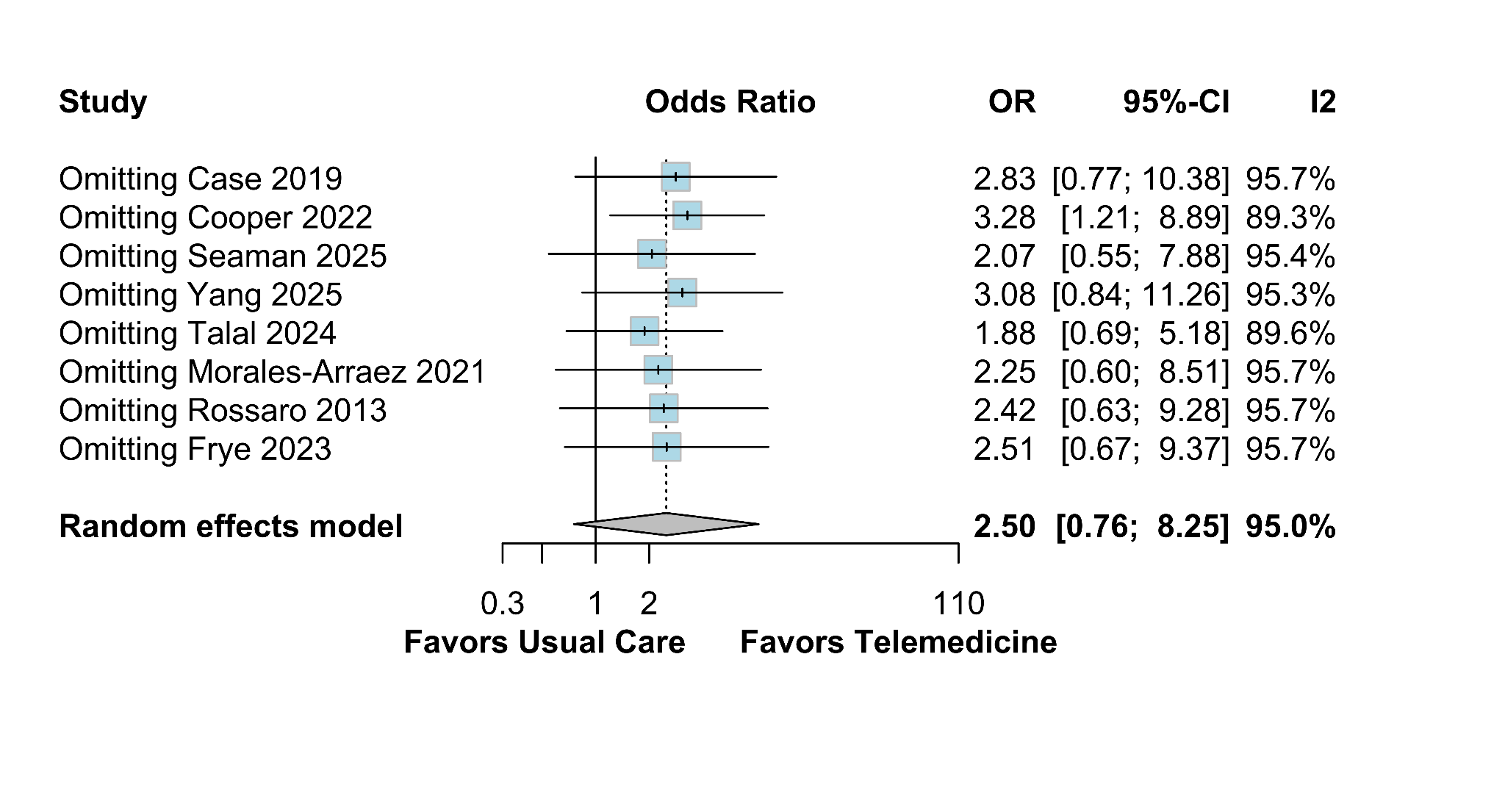

Supplement: Supplementary file 9 — Figure S9: Leave‐one‐out sensitivity analysis for treatment completion comparing synchronous telemedicine and in‐person care. [file JVH-33-0-s014.docx]

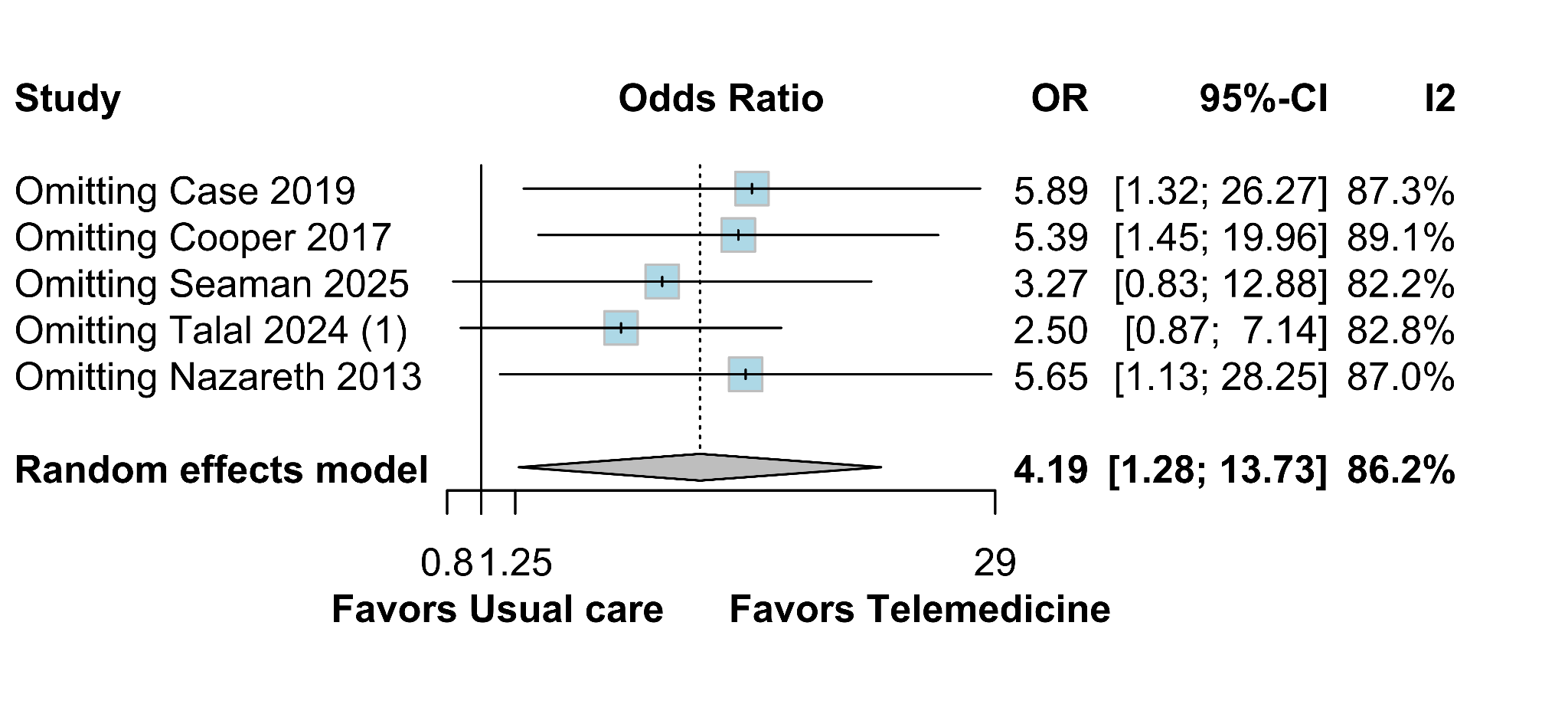

Supplement: Supplementary file 10 — Figure S10: Leave‐one‐out sensitivity analysis for sustained virologic response in rural settings compared with non‐rural settings. [file JVH-33-0-s022.docx]

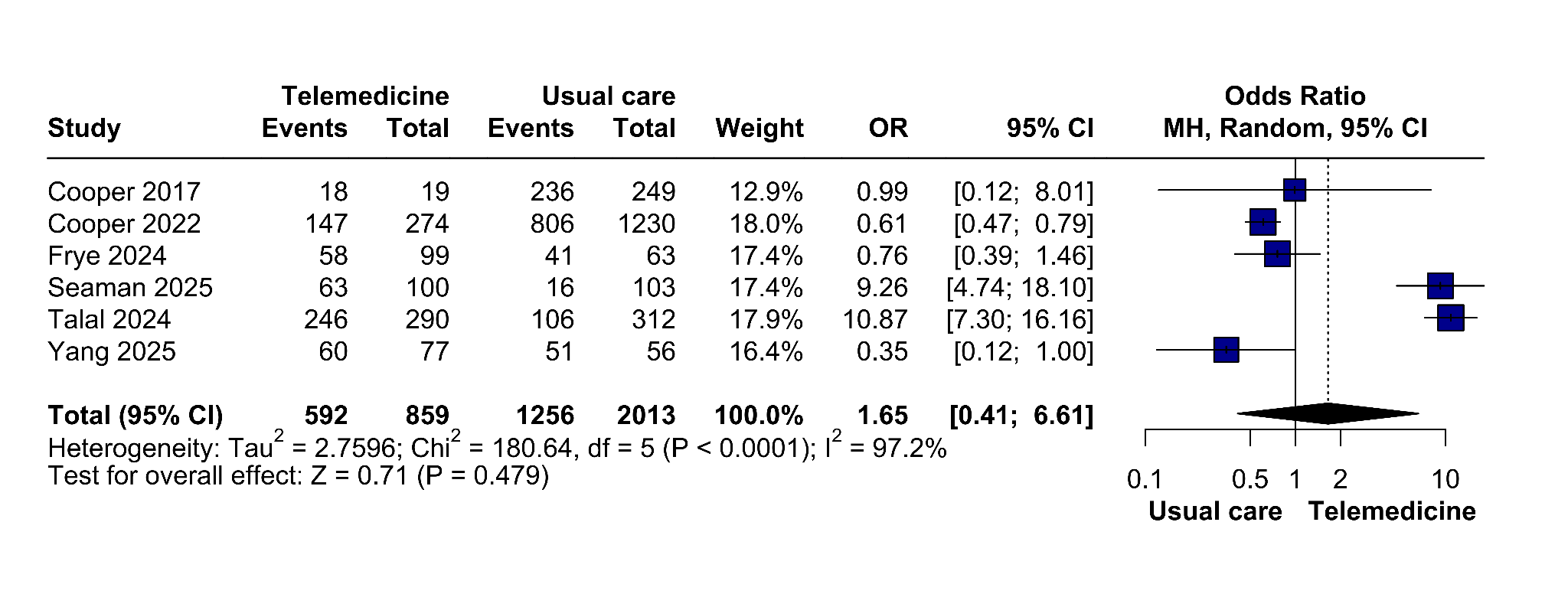

Supplement: Supplementary file 11 — Figure S11: Forest plot of sustained virologic response in studies of direct‐acting antiviral therapy comparing synchronous telemedicine and in‐person care. [file JVH-33-0-s017.docx]

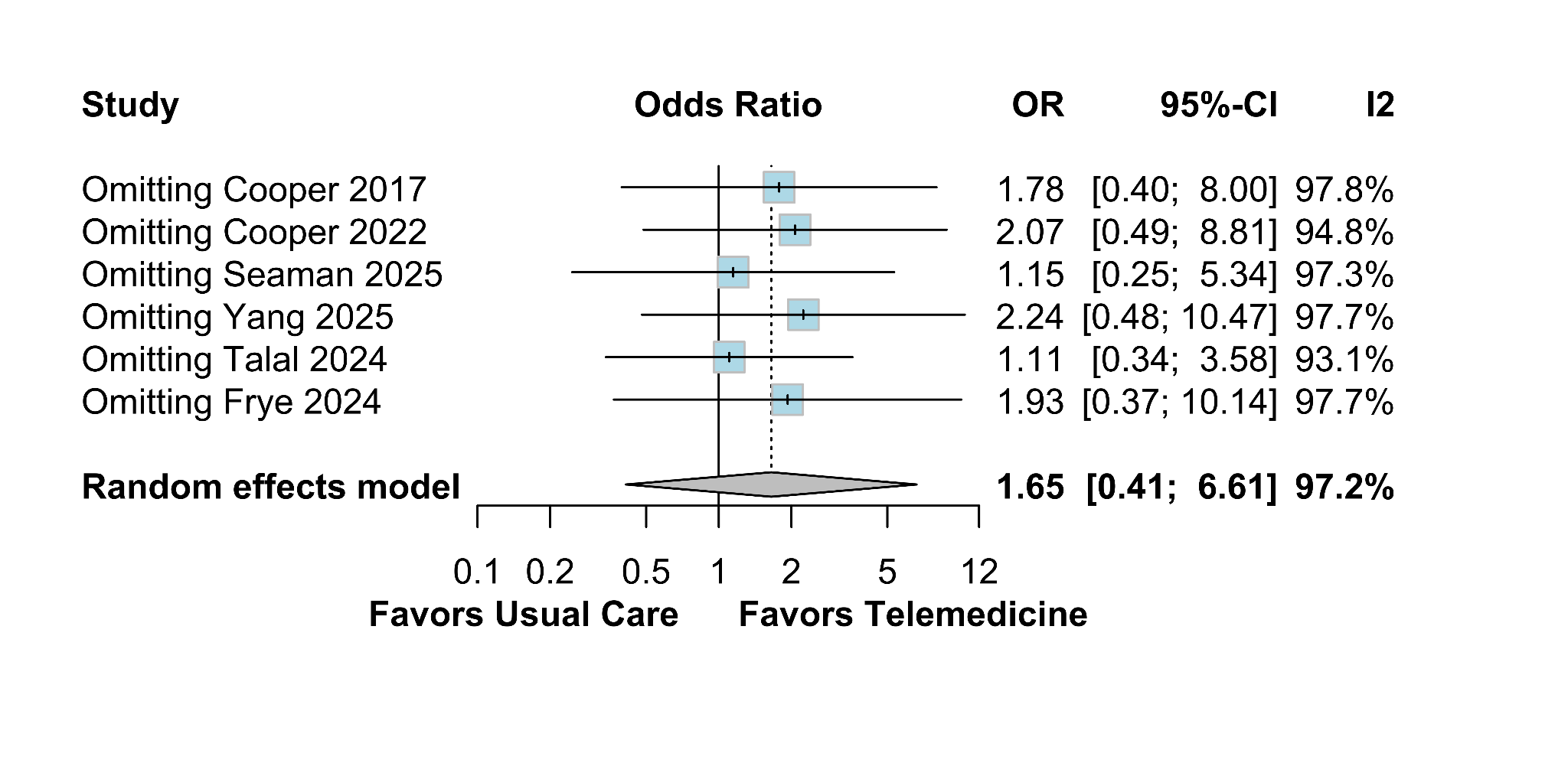

Supplement: Supplementary file 12 — Figure S12: Leave‐one‐out sensitivity analysis for sustained virologic response in studies of direct‐acting antiviral therapy comparing synchronous telemedicine and in‐person care. [file JVH-33-0-s001.docx]

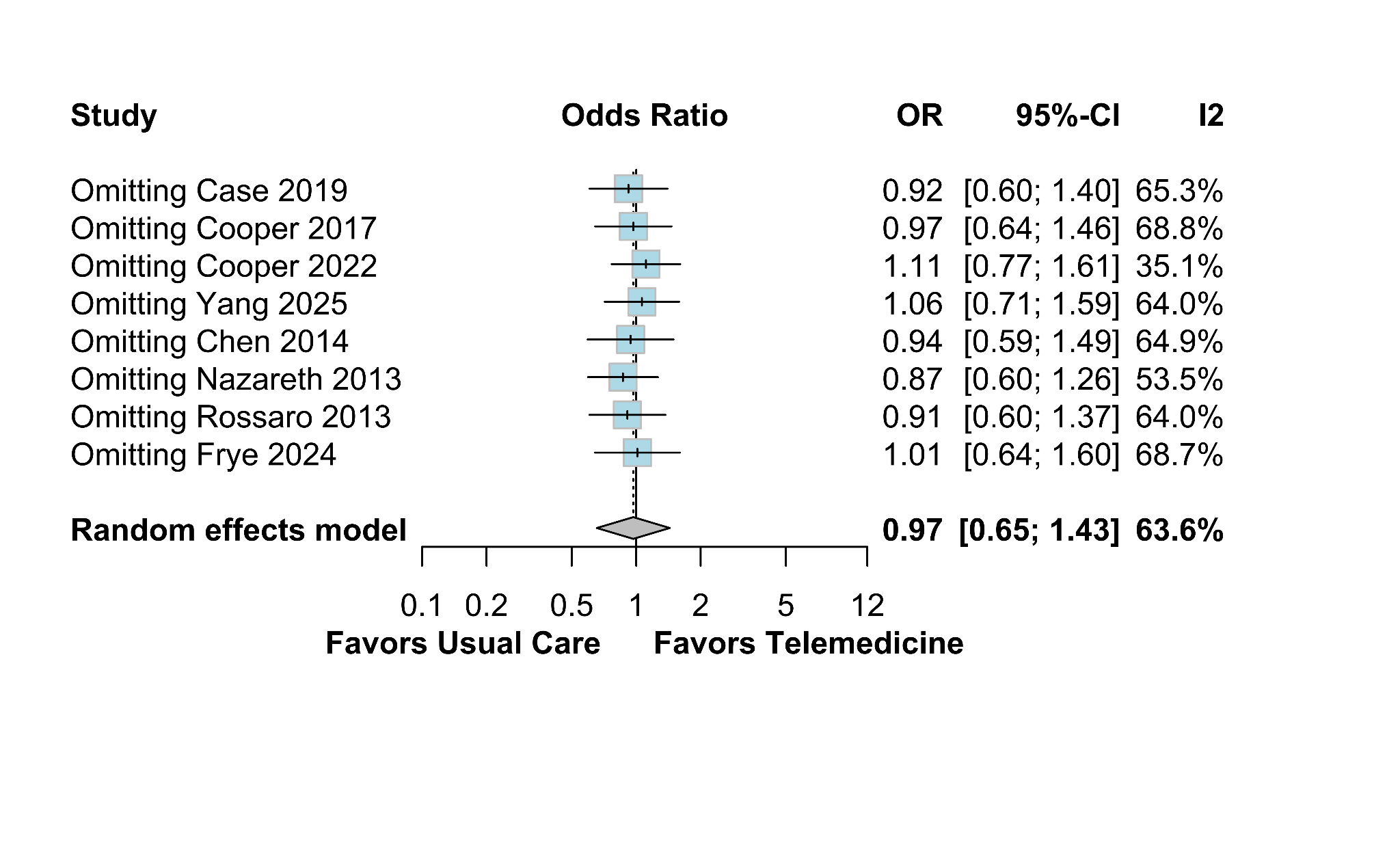

Supplement: Supplementary file 13 — Figure S13: Sensitivity analysis for sustained virologic response in observational studies comparing synchronous telemedicine and in‐person care. [file JVH-33-0-s012.docx]

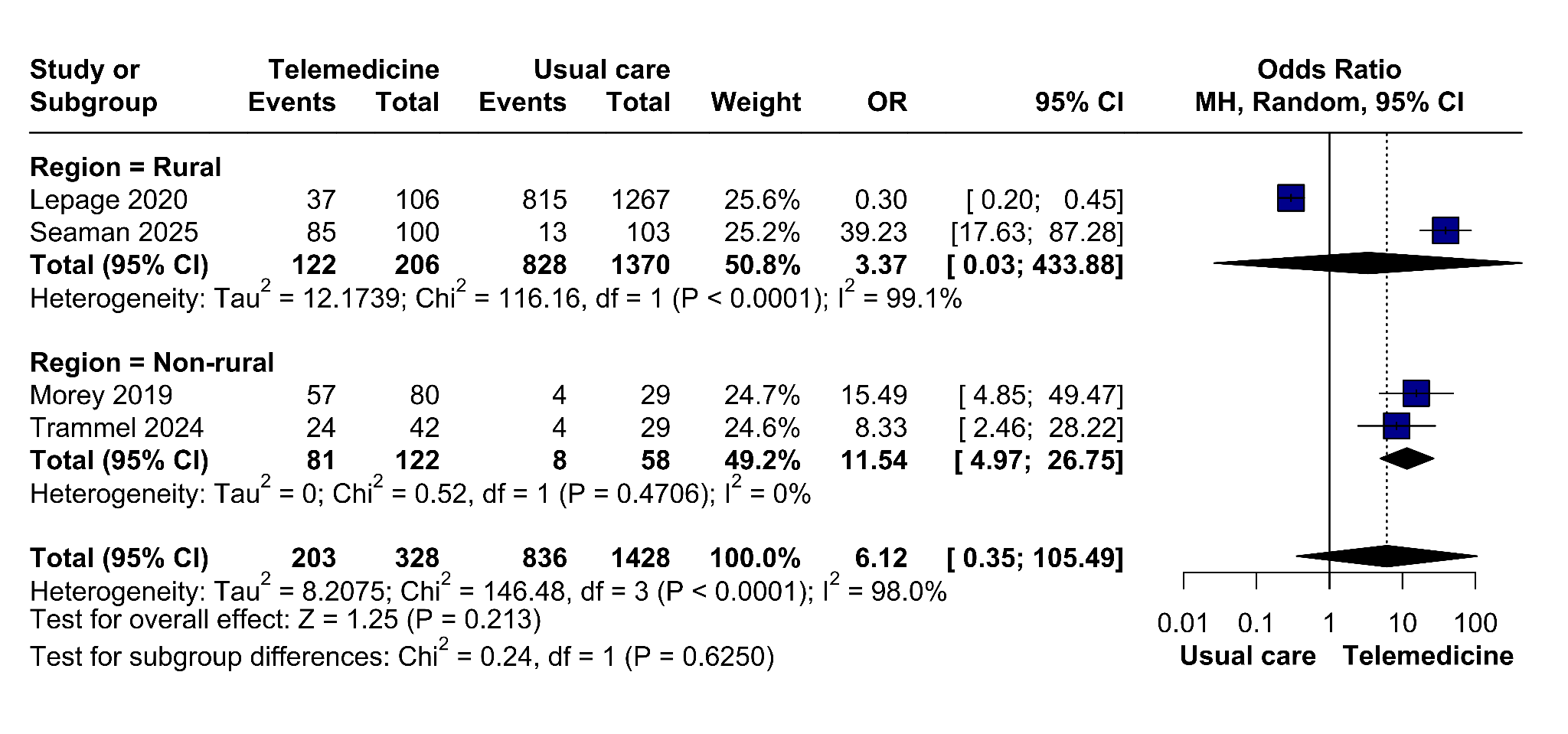

Supplement: Supplementary file 14 — Figure S14: Subgroup analysis of treatment initiation in rural settings compared with non‐rural settings. [file JVH-33-0-s015.docx]

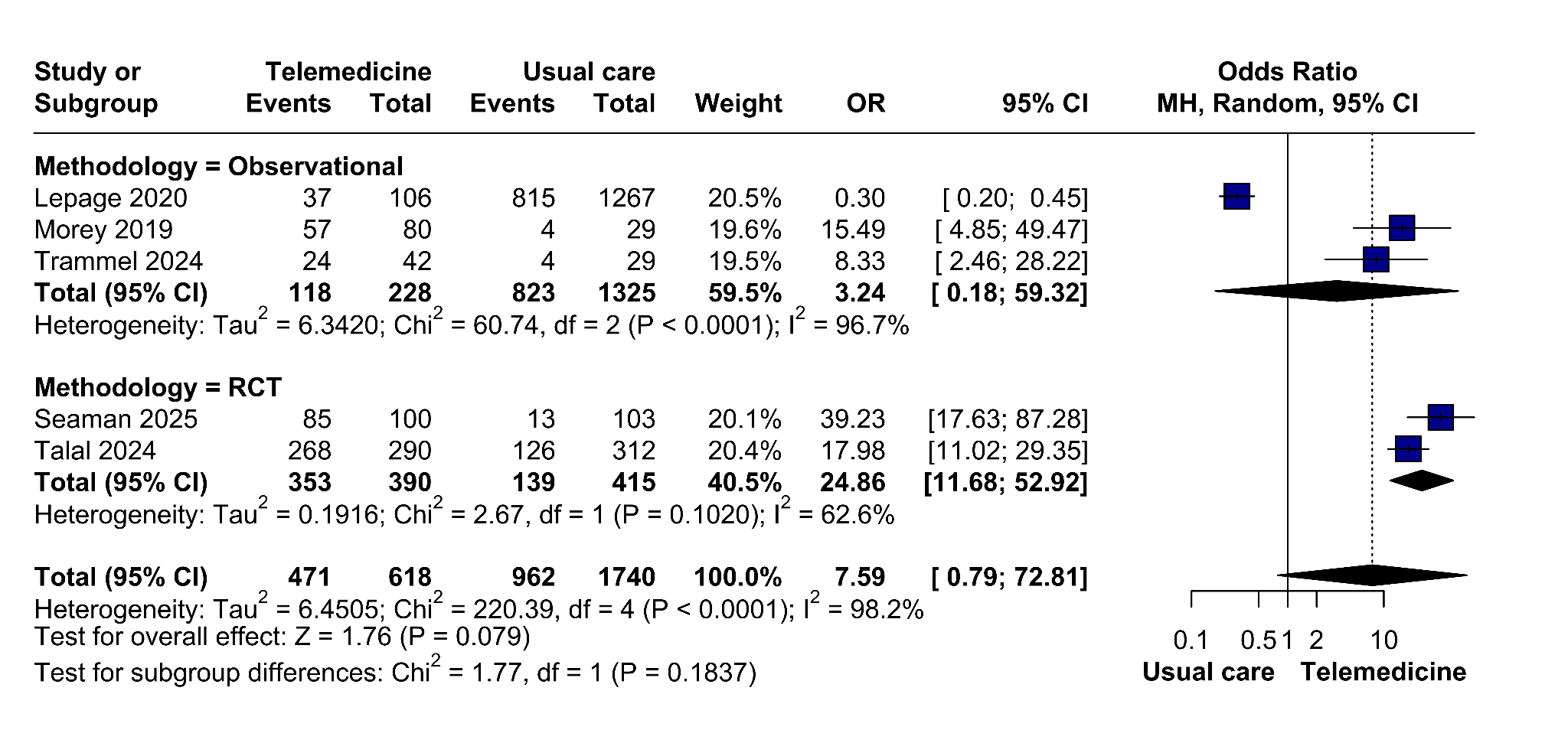

Supplement: Supplementary file 15 — Figure S15: Subgroup analysis of treatment initiation in randomised controlled trials compared with observational studies. [file JVH-33-0-s026.docx]

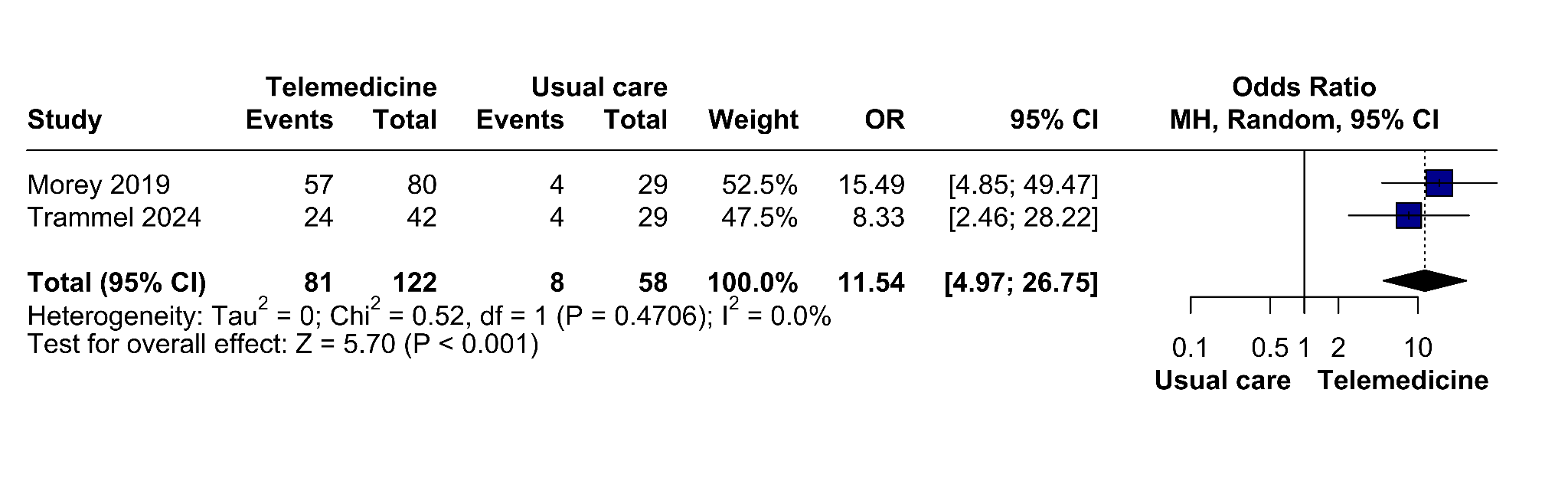

Supplement: Supplementary file 16 — Figure S16: Subgroup analysis of treatment initiation in studies of direct‐acting antiviral therapy in rural settings compared with non‐rural settings. [file JVH-33-0-s007.docx]

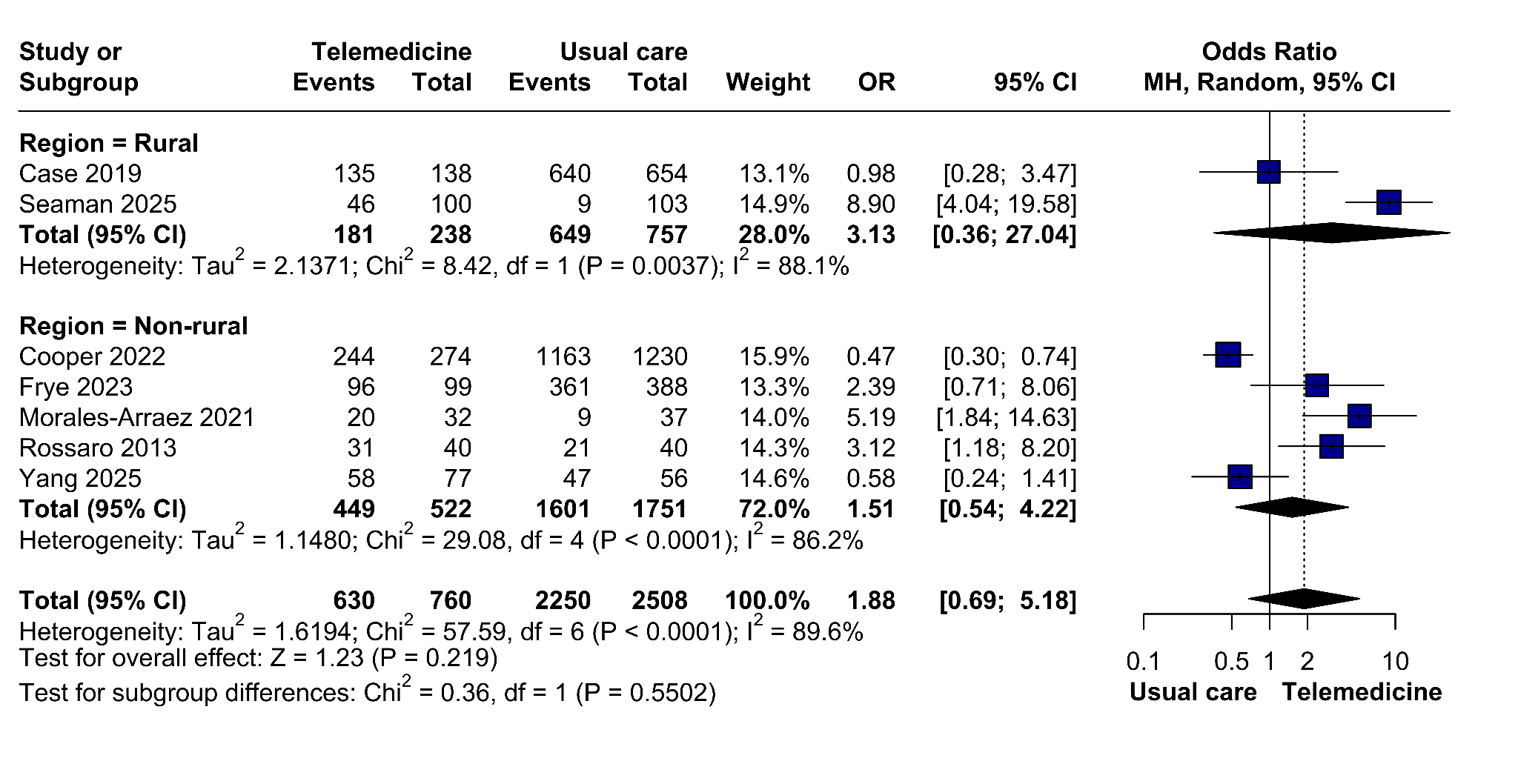

Supplement: Supplementary file 17 — Figure S17: Subgroup analysis of treatment completion in rural settings compared with non‐rural settings. [file JVH-33-0-s005.docx]

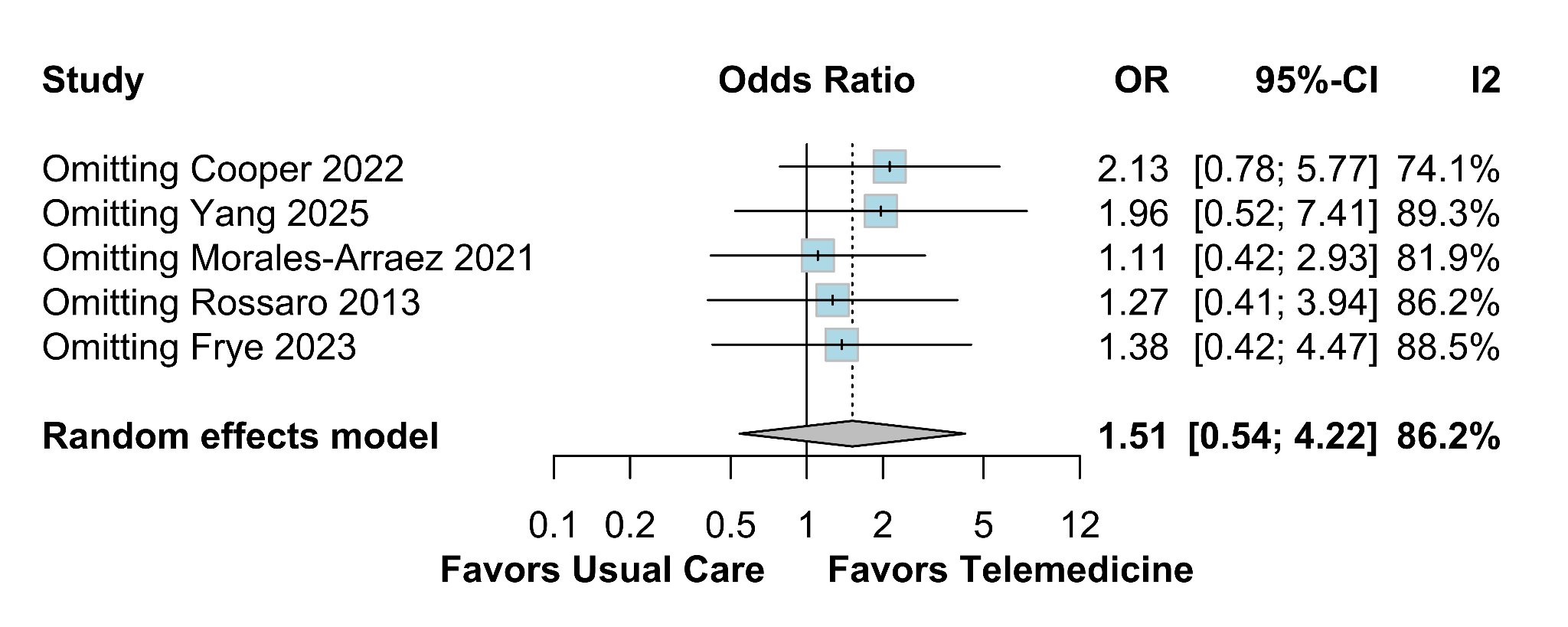

Supplement: Supplementary file 18 — Figure S18: Leave‐one‐out sensitivity analysis for treatment completion in non‐rural settings. [file JVH-33-0-s009.docx]

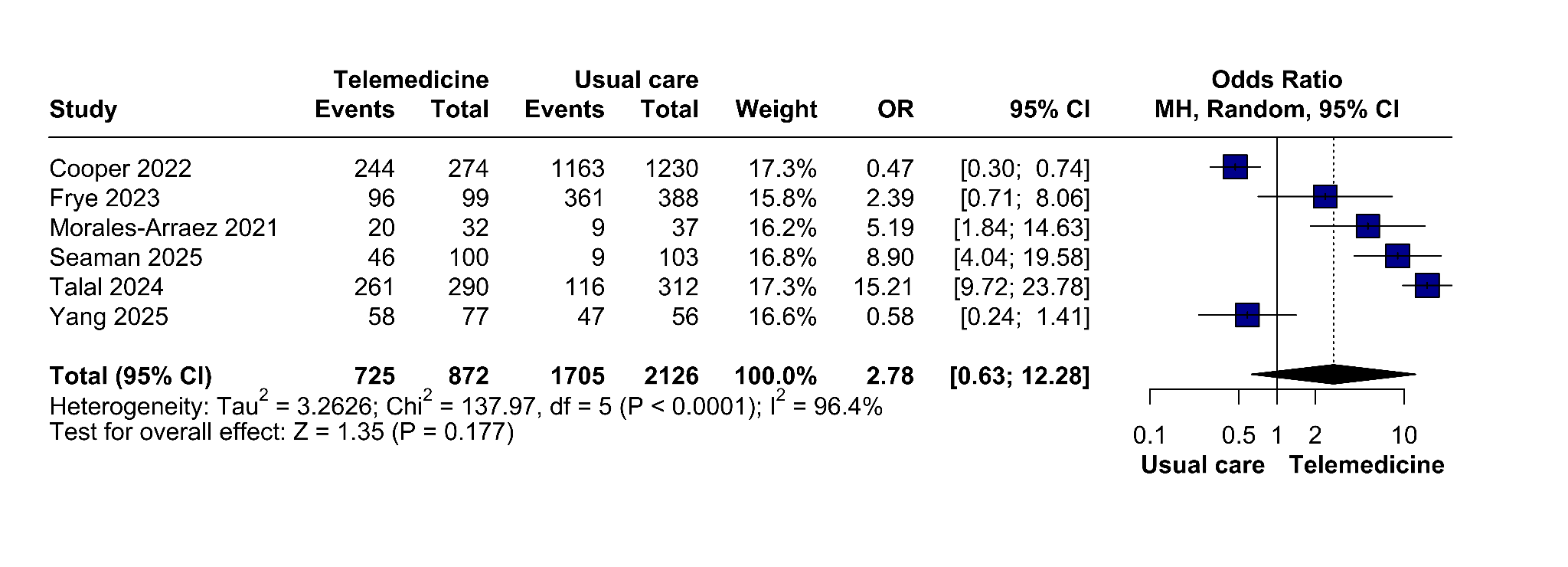

Supplement: Supplementary file 19 — Figure S19: Subgroup analysis of treatment completion in studies of direct‐acting antiviral therapy comparing synchronous telemedicine and in‐person care. [file JVH-33-0-s006.docx]

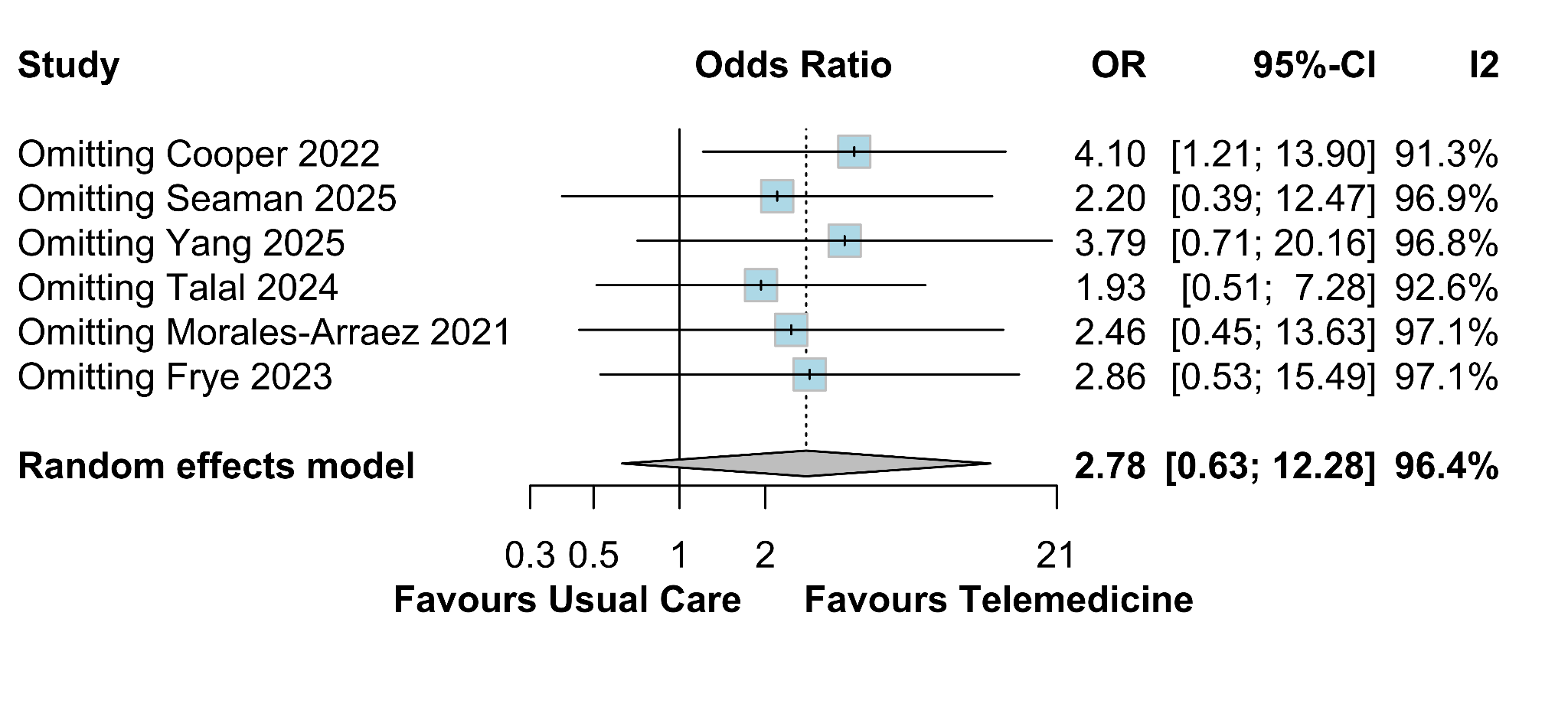

Supplement: Supplementary file 20 — Figure S20: Leave‐one‐out sensitivity analysis for treatment completion in studies of direct‐acting antiviral therapy comparing synchronous telemedicine and in‐person care. [file JVH-33-0-s018.docx]

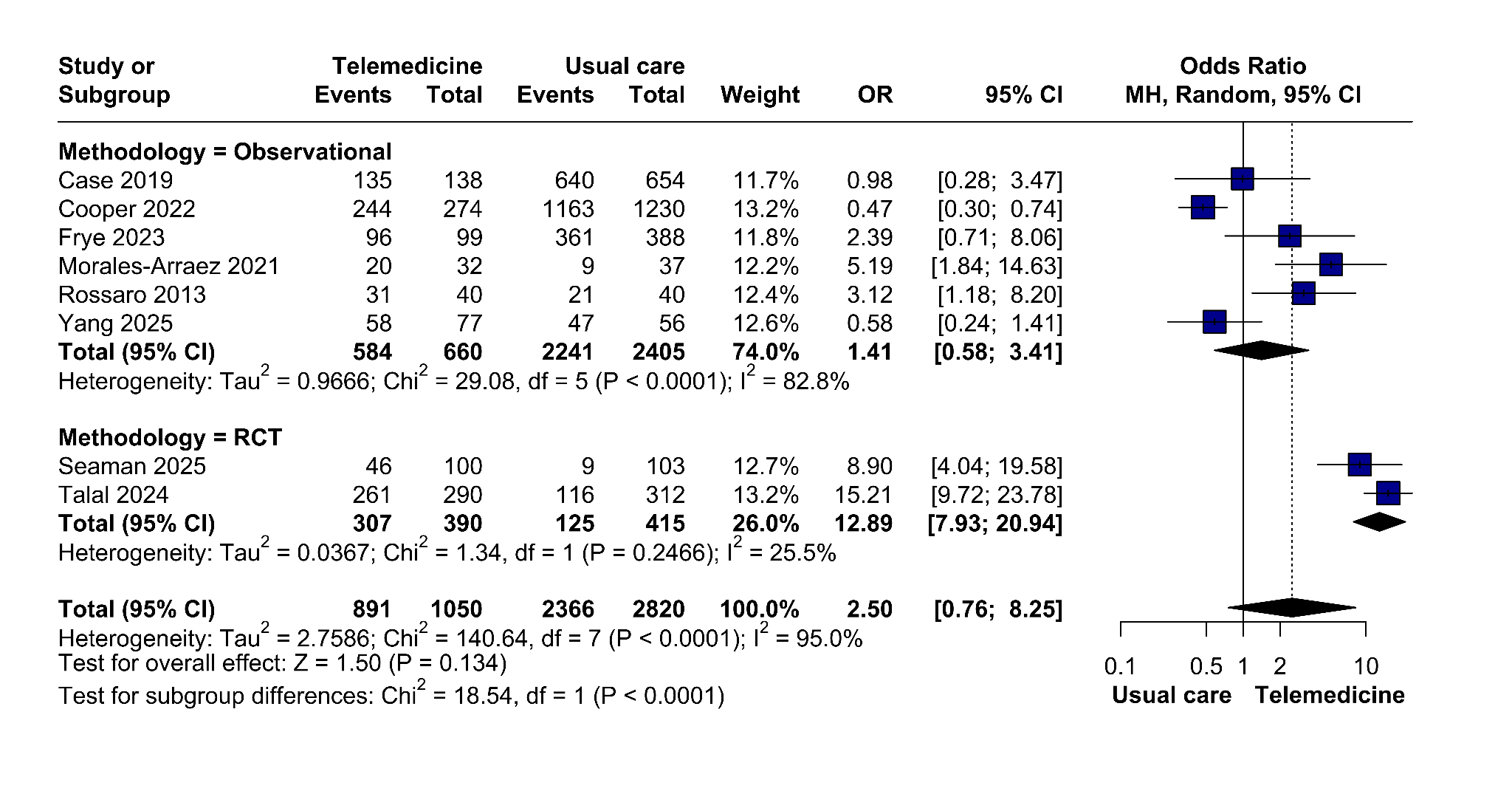

Supplement: Supplementary file 21 — Figure S21: Subgroup analysis of treatment completion in randomised controlled trials compared with observational studies. [file JVH-33-0-s021.docx]

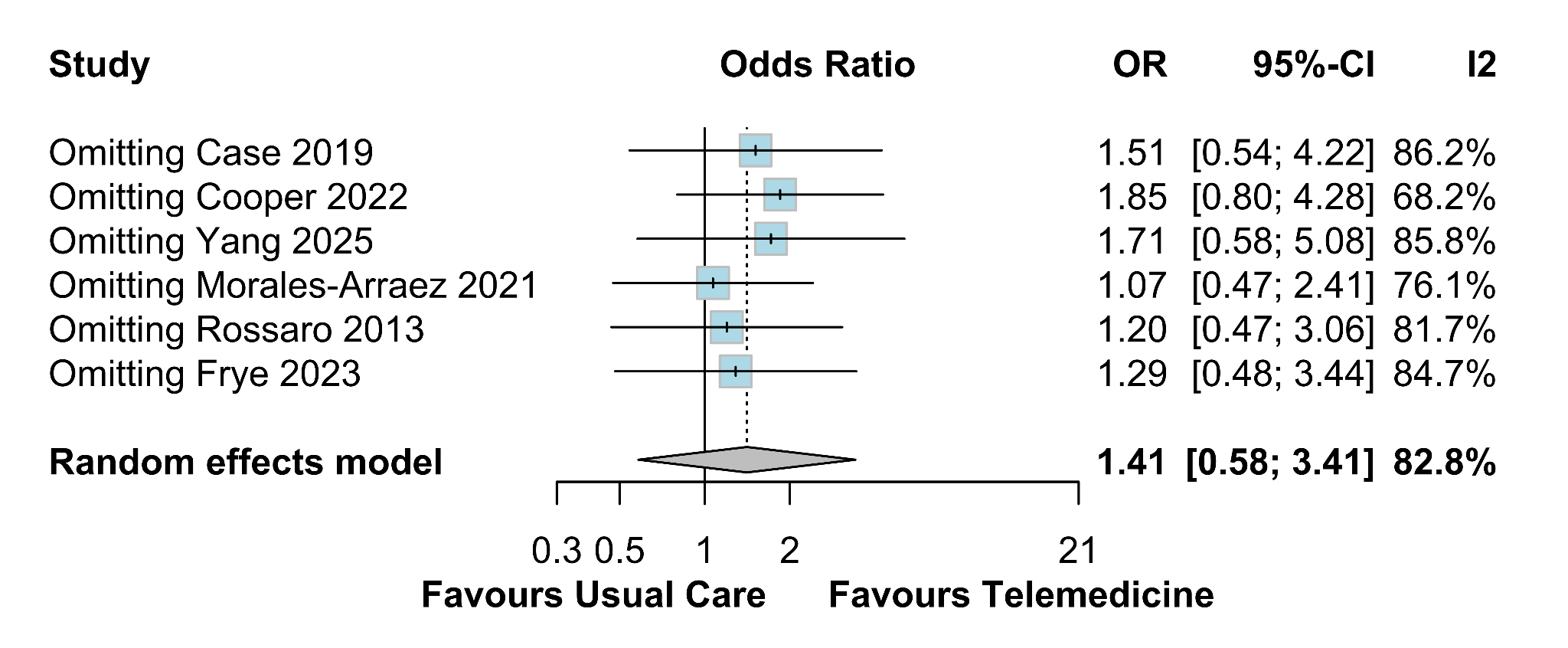

Supplement: Supplementary file 22 — Figure S22: Sensitivity analysis for treatment completion in observational studies comparing synchronous telemedicine and in‐person care. [file JVH-33-0-s013.docx]

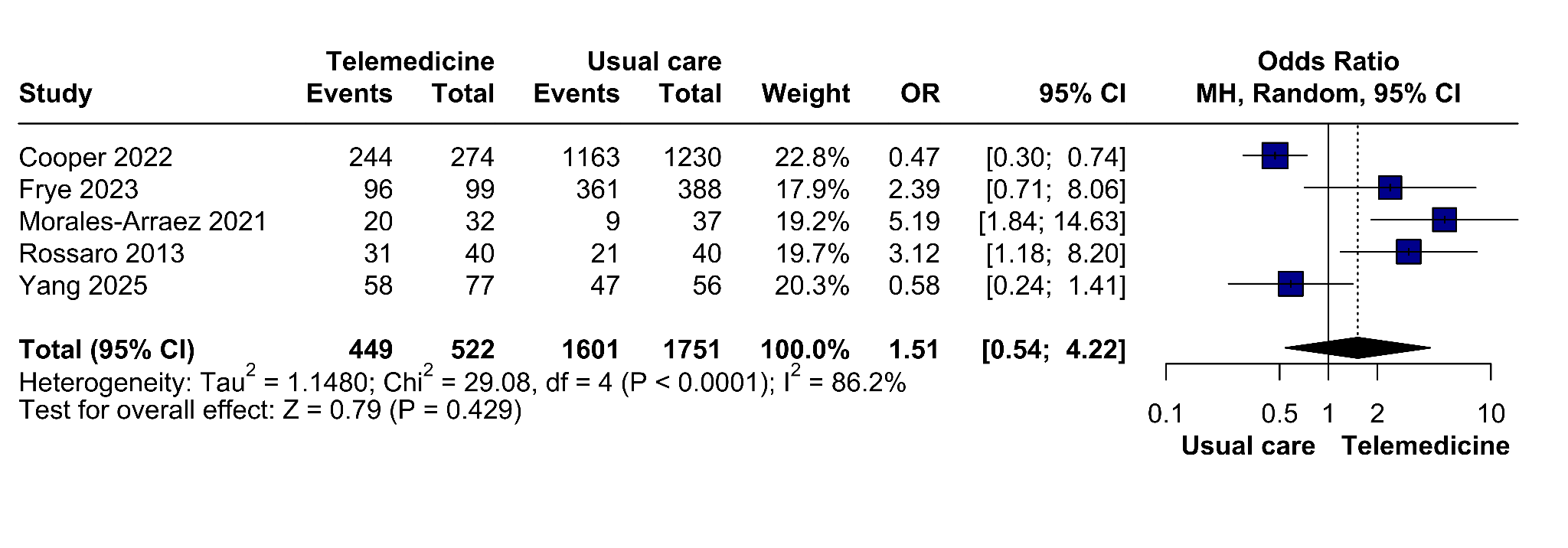

Supplement: Supplementary file 23 — Figure S23: Subgroup analysis of treatment completion in studies of direct‐acting antiviral therapy in rural settings compared with non‐rural settings. [file JVH-33-0-s004.docx]
