# Supplementary material for: Synchronous Telemedicine Versus In‐Person Care in Hepatitis C Treatment: A Systematic Review and Meta‐Analysis
Source: J Viral Hepat. 2026 Jan 28;33(3):e70144. doi: 10.1111/jvh.70144 (PMC12848981; doi:10.1111/jvh.70144)
Supplement: Supplementary file 25 — Table S2: Complete electronic search strategy for all databases. [file JVH-33-0-s024.docx]

Date: 25/06/2025

| Database | Search Strategy | Results |
| --- | --- | --- |
| PubMed | (emedicine OR "tele based" OR "video based" OR "web based" OR "phone based" OR "mobile based" OR "app based" OR "audio based" OR "voice based" OR telemedicine OR "tele medicine" OR telehealth OR "tele health" OR telecare OR "tele care" OR telecommunicat* OR "tele communicat*" OR teleconferenc* OR "tele conferenc*" OR teleconsultat* OR "tele consultat*" OR telemonitor* OR "tele monitor*" OR teletherap* OR "tele therap*" OR telerehab* OR "tele rehab*" OR ehealth OR "e-health" OR mhealth OR "m-health" OR "remote health" OR "remote care" OR "remote medicine" OR "remote communicat*" OR "remote therap*" OR "remote consult*" OR "remote conference*" OR "video conferenc*" OR videoconferenc* OR "digital health" OR "digital care") AND  (HCV OR "hepatitis C") | 456 |
| Embase | (emedicine:ti,ab,kw OR 'tele based':ti,ab,kw OR 'video based':ti,ab,kw OR 'web based':ti,ab,kw OR 'phone based':ti,ab,kw OR 'mobile based':ti,ab,kw OR 'app based':ti,ab,kw OR 'audio based':ti,ab,kw OR 'voice based':ti,ab,kw OR telemedicine:ti,ab,kw OR 'tele medicine':ti,ab,kw OR telehealth:ti,ab,kw OR 'tele health':ti,ab,kw OR telecare:ti,ab,kw OR 'tele care':ti,ab,kw OR telecommunicat*:ti,ab,kw OR 'tele communicat*':ti,ab,kw OR teleconferenc*:ti,ab,kw OR 'tele conferenc*':ti,ab,kw OR teleconsultat*:ti,ab,kw OR 'tele consultat*':ti,ab,kw OR telemonitor*:ti,ab,kw OR 'tele monitor*':ti,ab,kw OR teletherap*:ti,ab,kw OR 'tele therap*':ti,ab,kw OR telerehab*:ti,ab,kw OR 'tele rehab*':ti,ab,kw OR ehealth:ti,ab,kw OR 'e-health':ti,ab,kw OR mhealth:ti,ab,kw OR 'm-health':ti,ab,kw OR 'remote health':ti,ab,kw OR 'remote care':ti,ab,kw OR 'remote medicine':ti,ab,kw OR 'remote communicat*':ti,ab,kw OR 'remote therap*':ti,ab,kw OR 'remote consult*':ti,ab,kw OR 'remote conference*':ti,ab,kw OR 'video conferenc*':ti,ab,kw OR videoconferenc*:ti,ab,kw OR 'digital health':ti,ab,kw OR 'digital care':ti,ab,kw) AND ('hepatitis c'/exp OR hcv:ti,ab,kw OR 'hepatitis c':ti,ab,kw) | 840 |
| Cochrane | (emedicine OR (tele NEXT based) OR (video NEXT based) OR (web NEXT based) OR (phone NEXT based) OR (mobile NEXT based) OR (app NEXT based) OR (audio NEXT based) OR (voice NEXT based) OR telemedicine OR (tele NEXT medicine) OR telehealth OR (tele NEXT health) OR telecare OR (tele NEXT care) OR telecommunicat* OR (tele NEXT communicat*) OR teleconferenc* OR (tele NEXT conferenc*) OR teleconsultat* OR (tele NEXT consultat*) OR telemonitor* OR (tele NEXT monitor*) OR teletherap* OR (tele NEXT therap*) OR telerehab* OR (tele NEXT rehab*) OR ehealth OR e-health OR mhealth OR m-health OR (remote NEXT health) OR (remote NEXT care) OR (remote NEXT medicine) OR (remote NEXT communicat*) OR (remote NEXT therap*) OR (remote NEXT consult*) OR (remote NEXT conference*) OR (video NEXT conferenc*) OR videoconferenc* OR (digital NEXT health) OR (digital NEXT care)) | 95 |
