# Supplementary material for: Synchronous Telemedicine Versus In‐Person Care in Hepatitis C Treatment: A Systematic Review and Meta‐Analysis
Source: J Viral Hepat. 2026 Jan 28;33(3):e70144. doi: 10.1111/jvh.70144 (PMC12848981; doi:10.1111/jvh.70144)
Supplement: Supplementary file 26 — Table S3: Definitions of telemedicine, sustained virologic response, and inclusion criteria across the included studies. [file JVH-33-0-s010.docx]

| Study (year) | TM definition | SVR definition | Inclusion criteria |
| --- | --- | --- | --- |
| Case et al. (2019) | The model consisted of clinic-to-clinic video telemedicine between specialty providers at the Denver VA Medical Centre and patients located at one of six rural community-based outpatient clinics. | SVR was defined as undetectable HCV RNA at 10 weeks or more post-treatment. | Veterans aged 18 years or older, infected with any HCV genotype (1-6), who were non-cirrhotic or had compensated cirrhosis (Child Turcotte-Pugh A) |
| Chen et al. (2014) | The model was a telecare program offering 24-hour telephone consultation services via a health communication center. It included scheduled outbound calls from healthcare professionals and patient-initiated inbound calls. | SVR was defined as the absence of detectable HCV RNA at the end of the 72-week follow-up period, as measured by a PCR assay. | Patients aged 18 years or older who had never received interferon treatment, had at least 2000 copies/mL of HCV RNA, elevated alanine aminotransferase activity, and a liver biopsy consistent with chronic hepatitis C |
| Cooper et al. (2017) | TM patients were defined as having the majority of their clinic visits conducted using the Ontario Tele-Health Network's video and audio system. The system linked the patient and a remote site nurse to the HCV clinician and healthcare team at The Ottawa Hospital. | SVR was defined as being free of the virus 12 or more weeks after treatment completion. | Patients 18 years of age and older with chronic HCV infection were included in the analysis. |
| Cooper et al. (2022) | The COVID-19 pandemic-era model consisted of all scheduled clinic visits being conducted entirely via TM. Laboratory and diagnostic testing were scheduled at internal or external institutions based on patient convenience. | SVR was defined as the absence of HCV RNA by polymerase chain reaction testing at least 12 weeks after completion of drug therapy. | Patients 18 years of age and older who received a referral for an HCV direct-acting antiviral agent. |
| Cuadrado et al. (2021) | The model consisted of a videoconference between the specialist (at the hospital) and the inmate, who was accompanied by the prison doctor (at the prison). The study used the SARA Network, a secure system connecting European Public Administrations. | The study reported SVR rates but did not explicitly provide a time-based definition in the methods section. | Patients were inmates in the "El Dueso" prison with HCV viremia who were prescribed oral antivirals and had at least one telemedicine consultation. |
| Frye et al. (2023) | The TM option involved monthly video or telephone calls for treatment visits, with HCV medications being couriered to the patient's residence. On-treatment laboratory testing was not required | SVR was defined as undetectable HCV RNA levels 12 weeks after completing antiviral therapy. | All patients who initiated HCV treatment at the Grady Liver Clinic |
| Lepage et al. (2020) | TM was defined as any clinic visit using video and teleconference technology to link an HCV patient to the healthcare team. The service utilized The Ontario Telemedicine Health Network's equipment at geographically separate medical clinics. | SVR was defined as HCV RNA negativity at 12 weeks or more after the completion of HCV antiviral therapy. | Patients aged 18 years or older who had engaged with The Ottawa Hospital Viral Hepatitis Program for HCV care at least once. |
| Morales-Arraez et al. (2021) | The model used videoconferences between the hepatologist and the patient, who was assisted by staff at a drug treatment center. This was linked to a decentralized model where medication was delivered to and supervised by the drug treatment center staff. | The study reported SVR rates but did not explicitly provide a time-based definition in the methods section. | Patients aged 18 years or older attending drug treatment centers who were willing to participate, had a valid public health card, and provided signed informed consent |
| Morey et al. (2019) | The treatment pathway used consultant-led TM clinics with nurse-led prison in-reach. After an in-person workup by the nurse, a follow-up consultation was conducted via video link between the patient and the hepatology consultant. | SVR was defined as HCV RNA not detected 3 months post-treatment. | Incarcerated individuals at specific prisons in North East England who were found to be HCV RNA positive were offered an assessment for treatment. |
| Nazareth et al. (2013) | The model used videoconferencing for review and treatment of rural and remote patients by a hepatologist and nurse practitioner. The independent, nurse-led clinics included consultation, therapy initiation, patient education, and follow-up via bi-weekly videoconferences. | SVR was defined as undetectable HCV RNA at least 24 weeks after the end of therapy. | Patients aged 18 years or older with documented chronic HCV and compensated disease who were not pregnant or trying to become pregnant and agreed to use contraception. |
| Rossaro et al. (2013) | The model used real-time audio-visual interaction to connect a patient and their local primary care provider (who was physically present with the patient) with an off-site specialist via videoconference. | SVR was defined as an undetectable HCV RNA level 24 weeks after the completion of therapy | Treatment-naïve patients between the ages of 18 and 75 years with documented HCV infection via PCR, who were referred for treatment by a primary care provider. |
| Seaman et al. (2025) | The model was peer-assisted telemedicine (TeleHCV), where peer support specialists facilitated visits by bringing communication devices (phone, laptop, or tablet) to patients in rural settings. The peers also supported medication delivery and adherence, connecting patients to a remote clinical team at an academic medical center | HCV viral clearance was defined as an undetectable HCV RNA level at 12 weeks after treatment completion (SVR₁₂) for those who initiated treatment, and as an undetectable HCV RNA at 9 months post-randomization for those who did not initiate. | Individuals 18 years or older living in one of seven rural Oregon counties who had a detectable HCV RNA level and had injected drugs or used non-prescribed opioids within the past 90 days. |
| Talal et al. (2024) | The model was "facilitated telemedicine," where a health care staff member (case manager) facilitated an on-site video encounter between a patient at an opioid treatment program and an off-site hepatitis specialist. Medications were delivered and dispensed with the patient's daily methadone. | SVR was defined as an undetectable Hepatitis C virus (HCV) RNA level 12 weeks after treatment cessation | Patients aged 18 years or older with detectable HCV RNA, at least 6 months of active enrollment in an opioid treatment program, and insurance coverage for DAA. |
| Trammel et al. (2024) | The TM model included phone and computer-based visits with an Infectious Disease specialist. When a patient was unable to attend a virtual visit, the model also included an electronic chart review by the specialist, who then communicated treatment recommendations directly to the patient's obstetrician | A specific definition for Sustained Virologic Response (SVR) was not explicitly provided in the text. The study focused on postpartum follow-up and treatment initiation rates. | All pregnant patients with substance use disorders who were seen in the Center for Acceptance and Recovery clinic at a single academic center |
| Yang et al. (2025) | The "minimal monitoring" model consisted of entirely telephonic or video-based clinic visits during treatment, without on-treatment laboratory monitoring. This was supplemented by telephonic support from a clinical pharmacist practitioner and a nurse care coordinator. | SVR was defined as an HCV RNA level less than the assay's lower limit of quantitation after 12 weeks treatment cessation. | Treatment-naïve patients aged 18 years or older with HCV infection who received DAA therapy at a single center |

Abbreviations: COVID-19: Coronavirus Disease 2019; CTP: DAA: Direct-Acting Antiviral; HCV: Hepatitis C Virus; PCR: Polymerase Chain Reaction; RNA: Ribonucleic Acid; SARA Network: A secure system connecting European Public Administrations; SVR: Sustained Virologic Response; TM: Telemedicine;
